# Sample size determination for a binary response in a superiority clinical trial using a hybrid classical and Bayesian procedure

**DOI:** 10.1186/s13063-017-1791-0

**Published:** 2017-02-23

**Authors:** Maria M. Ciarleglio, Christopher D. Arendt

**Affiliations:** 1 Department of Biostatistics, Yale University School of Public Health, 60 College Street, New Haven, 06510 CT USA; 2 Clinical Epidemiology Research Center, VA Cooperative Studies Program, 950 Campbell Avenue, Bldg 35A, West Haven, 06516 CT USA; 3 Air Force Office of Scientific Research, 875 N Randolph Street, Arlington, 22203 VA USA

**Keywords:** Sample size, Clinical trial, Proportions, Binary endpoint, Conditional expected power, Hybrid classical-Bayesian

## Abstract

**Background:**

When designing studies that have a binary outcome as the primary endpoint, the hypothesized proportion of patients in each population experiencing the endpoint of interest (i.e., *π*_1_,*π*_2_) plays an important role in sample size and power calculations. Point estimates for *π*_1_ and *π*_2_ are often calculated using historical data. However, the uncertainty in these estimates is rarely addressed.

**Methods:**

This paper presents a hybrid classical and Bayesian procedure that formally integrates prior information on the distributions of *π*_1_ and *π*_2_ into the study’s power calculation. Conditional expected power (CEP), which averages the traditional power curve using the prior distributions of *π*_1_ and *π*_2_ as the averaging weight conditional on the presence of a positive treatment effect (i.e., *π*_2_>*π*_1_), is used, and the sample size is found that equates the pre-specified frequentist power (1−*β*) and the conditional expected power of the trial.

**Results:**

Notional scenarios are evaluated to compare the probability of achieving a target value of power with a trial design based on traditional power and a design based on CEP. We show that if there is uncertainty in the study parameters and a distribution of plausible values for *π*_1_ and *π*_2_, the performance of the CEP design is more consistent and robust than traditional designs based on point estimates for the study parameters. Traditional sample size calculations based on point estimates for the hypothesized study parameters tend to underestimate the required sample size needed to account for the uncertainty in the parameters. The greatest marginal benefit of the proposed method is achieved when the uncertainty in the parameters is not large.

**Conclusions:**

Through this procedure, we are able to formally integrate prior information on the uncertainty and variability of the study parameters into the design of the study while maintaining a frequentist framework for the final analysis. Solving for the sample size that is necessary to achieve a high level of CEP given the available prior information helps protect against misspecification of hypothesized treatment effect and provides a substantiated estimate that forms the basis for discussion about the study’s feasibility during the design phase.

## Background

When designing a study that has a binary outcome as the primary endpoint, the hypothesized proportion of patients in each population experiencing the endpoint of interest (i.e., *π*_1_,*π*_2_) plays an important role in sample size determination. In a two-arm study comparing two independent proportions, |*π*_2_−*π*_1_| represents the true hypothesized difference between groups, sometimes also known as the minimal relevant difference [1]. While the treatment effect may also be parameterized equivalently using an odds ratio or relative risk, when appropriate, the most frequently used sample size formula expresses the treatment effect using the difference between groups [2, 3]. In the case of proportions, the variance of the difference depends on the individual hypothesized values for the population parameters *π*_1_ and *π*_2_ under the alternative hypothesis. Thus, the sample size required to detect a particular difference of interest is affected by both the magnitude of the difference and the individual hypothesized values.

Traditional sample size formulas incorporate beliefs about *π*_1_ and *π*_2_ through single point estimates [1]. However, there is often uncertainty in these hypothesized proportions and, thus, a distribution of plausible values that should be considered when determining sample size. Misspecification of these hypothesized proportions in the sample size calculation may lead to an underpowered study, or one that has a low probability of detecting a smaller and potentially clinically relevant difference when such a difference exists [4]. Alternatively, if there is strong evidence in favor of a large difference, a study may be overpowered to detect a small hypothesized difference. Thus, a method for determining sample size that formally uses prior information on the distribution of study design parameters can mitigate the risk that the power calculation will be overly optimistic or overly conservative.

Similar difficulty surrounding the choice of study parameters for a continuous endpoint with known variance [5] and for a continuous endpoint with unknown variance [6] has been discussed previously. We have presented methods that formally incorporate the distribution of prior information on both the treatment effect and the variability of the endpoint into sample size determination. In this paper, we extend these methods to a binary endpoint by using a “hybrid classical and Bayesian” [7] technique based on conditional expected power (CEP) [8] to account for the uncertainty in study parameters *π*_1_ and *π*_2_ when determining the sample size of a superiority clinical trial. Unlike traditional power, which is calculated assuming the truth of a point alternative hypothesis (*π*_2_−*π*_1_=*Δ*_*A*_) for given values of *π*_1_ and *π*_2_, CEP conditions on the truth of a composite alternative of superiority (e.g., *π*_2_−*π*_1_>0 or *π*_2_>*π*_1_). CEP formally incorporates available prior information on both *π*_1_ and *π*_2_ into the power calculations by averaging the traditional power curve using the product of the prior distribution of *π*_1_ and the *conditional* prior distribution of *π*_2_,*p*(*π*_2_ | *π*_2_>*π*_1_), as the averaging weight. Based on the available prior information, the sample size that yields the desired level of CEP can be used when estimating the required sample size of the study.

While there has been much research in the area of Bayesian sample size determination [9–12], the hybrid classical and Bayesian method presented here aligns more with the ideas found in traditional frequentist sample size determination. Unlike traditional frequentist methods, however, we do not assume that the true parameters under the alternative hypothesis are known. This assumption rarely holds; typically, parameter values are estimated from early phase or pilot studies, studies of the intervention in different populations, or studies of similar agents in the current population [13, 14]. Thus, there is uncertainty surrounding the estimation of these population parameters and natural prior distributions of plausible values of these parameters that should be incorporated into the assessment of a trial’s power. Our method incorporates knowledge on the magnitude and uncertainty in the parameters into the traditional frequentist notion of power through explicit prior distributions on these unknown parameters to give CEP. As discussed in the “Methods” Section, CEP is not only well behaved, but it allows us to maintain a definition of power that intuitively converges to the traditional definition. Bayesian methodology is used only during the study design to allow prior information, through the prior distributions, to inform a choice for the sample size. Traditional type I and type II error rates, which have been accepted in practice, are maintained, and inferences are based on the likelihood of the data. The probability of achieving a target value of power using this method is compared to the performance of a traditional design. It is our hope that this formal method for incorporating prior knowledge into the study design will form the basis of thoughtful discussion about the feasibility of the study in order to reduce the number of poorly designed, underpowered studies that are conducted.

## Methods

### CEP for dichotomous outcome

Suppose that the study endpoint is dichotomous so that the probability (risk) of experiencing the event of interest in group 2 (the experimental treatment group), *π*_2_, is compared to that in group 1 (the control group), *π*_1_. The responses (i.e., the number of successes) in each group follow a binomial distribution. Assume that after *n* observations in each independent group or *N*=2*n* total observations, the two-sample *Z*-test of proportions is performed to test the null hypothesis *H*_0_:*π*_2_=*π*_1_ (i.e., *π*_2_−*π*_1_=*Δ*=0) versus the two-sided alternative hypothesis *H*_1_:*π*_2_≠*π*_1_ (i.e., *π*_2_−*π*_1_=*Δ*≠0), where *π*_2_>*π*_1_ indicates benefit of the experimental treatment over the control. The test is based on the test statistic *T*=*p*_2_−*p*_1_, or the difference in the proportion of successes in each sample. Under *H*_0_:*π*_2_=*π*_1_=*π*,*T* · ∼ *N*(0,*σ*_0_) in large samples, where *σ*_0_ is the *standard deviation* of the normal distribution. Assuming equal sample sizes *n* in each group gives $\sigma _{0} = \sqrt {2 \pi (1-\pi)/n }$, where *π*=(*π*_1_+*π*_2_)/2. In this setting, *H*_0_ is rejected at the *α*-level of significance if $|T| \geq z_{{}_{1-\alpha /2}} \, \hat {\sigma }_{0}$, where $\phantom {\dot {i}\!}z_{{}_{1-\alpha /2}}$ is the critical value for lower tail area 1−*α*/2 of the standard normal distribution and *π* is estimated by *p*=(*p*_1_+*p*_2_)/2 in $\hat {\sigma }_{0}$. A positive conclusion, *D*_1_, occurs if $Z = T/\hat {\sigma }_{0} \geq z_{{}_{1-\alpha /2}}$.

**Fig. 1:**
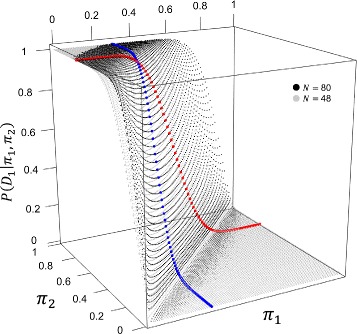
Traditional power surfaces when hypothesized values of *π*_1_=0.3 and *π*_2_=0.7 for *N*=48 and *N*=80

Under $H_{1}: \pi _{2}-\pi _{1} = \Delta _{A}, T \overset {\cdot }{\sim } N(\Delta _{A}, \sigma _{1})$, where $\sigma _{1} = \sqrt {(\pi _{2} (1-\pi _{2}) + \pi _{1} (1-\pi _{1}))/n}$. Thus, the traditional power of this test to detect the hypothesized difference corresponding to values of *π*_1_ and *π*_2_ under *H*_1_ is

1 $$ \begin{aligned} {}P(D_{1} \,|\, \pi_{1}, \pi_{2}) = \Phi\left[ \frac{\sqrt{N} \, |\pi_{2}-\pi_{1}| - 2 z_{{}_{1-\alpha/2}} \sqrt{\pi(1-\pi)}}{\sqrt{2 \pi_{2} (1-\pi_{2}) + 2 \pi_{1} (1-\pi_{1})}} \right] \!= 1-\beta,  \end{aligned}  $$

where *Φ*[ ·] is the standard normal cumulative distribution function. Since the traditional power curve is discontinuous at *π*_2_=*π*_1_ for a two-sided test, we assume a successful outcome or *π*_2_>*π*_1_ when calculating power; thus, |*π*_2_−*π*_1_|=*π*_2_−*π*_1_ in (1). One may plot the power function for fixed *N* and *π*_1_ over values of *π*_2_ or equivalently over values of *π*_2_−*π*_1_ to give the traditional power curve. Figure 1 shows the traditional power surfaces for *N*=48 and for *N*=80 with hypothesized values of *π*_2_=0.7 and *π*_1_=0.3. Power curves for fixed *π*_2_=0.7 and variable *π*_1_ and for fixed *π*_1_=0.3 and variable *π*_2_ are highlighted. Sample size is chosen to give high traditional power (e.g., 0.80≤1−*β*≤0.90) to detect an effect at least as large as the hypothesized difference for *π*_2_ and *π*_1_ by solving (1) for *N* [2]:

2 $$ \begin{aligned} {}N = \left[\frac{2 z_{{}_{1-\alpha/2}} \sqrt{\pi(1-\pi)} + z_{{}_{1-\beta}} \sqrt{2 \pi_{2} (1-\pi_{2}) + 2 \pi_{1} (1-\pi_{1})}}{\pi_{2}-\pi_{1}} \right]^{2}.  \end{aligned}  $$

The traditional power curve does not account for the uncertainty associated with the unknown population parameters *π*_2_ and *π*_1_ and does not indicate if the planned sample size is adequate given this uncertainty. Average or expected power (EP) was developed as a way to use the distribution of prior beliefs about the unknown parameters to provide an overall predictive probability of a positive conclusion [8, 9, 15–24]. EP, also known as assurance [20], probability of study success [23], or Bayesian predictive power [24], averages the traditional power curve using the prior distributions for the unknown parameters to weight the average *without restricting the prior distributions to assume treatment superiority*. In the case of a binomial response, assuming *π*_1_ and *π*_2_ are independent yields a special case of the general multivariate formulation which allows the joint distribution *p*(*π*_1_,*π*_2_) to be factored into the product of the two prior distributions *p*(*π*_1_) and *p*(*π*_2_). Thus, the traditional power curve *P*(*D*_1_ | *π*_2_,*π*_1_) is averaged using the product of the prior distributions for *π*_2_ and *π*_1_,*p*(*π*_2_) and *p*(*π*_1_), respectively, as the averaging weight [8], which gives the following formulation for EP:

$$P(D_{1}) = \int\limits_{\pi_{1}} \int\limits_{\pi_{2}} \, P(D_{1} \,|\, \pi_{1}, \pi_{2}) \, p(\pi_{2}) \, p(\pi_{1}) \, d\pi_{2} \, d\pi_{1}. $$

Expected power *conditional on the experimental treatment’s superiority*, *π*_2_>*π*_1_, is known as conditional expected power (CEP) [8]. Unlike EP, CEP is found by using the conditional prior distribution for *π*_2_,*p*(*π*_2_ | *π*_2_>*π*_1_), in the averaging weight. Since this conditional prior is now dependent on *π*_1_ and equals zero when *π*_2_≤*π*_1_, to ensure integration to 1 when *P*(*π*_1_>*π*_2_)>0, the conditional prior is scaled by the normalization factor *P*(*π*_2_>*π*_1_)^−1^, or the inverse probability of the experimental treatment’s superiority. This gives the following formulation for CEP:

3 $$ \begin{aligned} {}P(D_{1} | \pi_{2} > \pi_{1}) &\,=\, \int\limits_{\pi_{1}} \int\limits_{\pi_{2}>\pi_{1}} \!P(D_{1} | \pi_{1}, \pi_{2}) p(\pi_{2} | \pi_{2}>\pi_{1}) p(\pi_{1}) \, d\pi_{2} \, d\pi_{1}\\[-3pt] &=\! \frac{1}{P(\pi_{2}\!\!>\!\!\pi_{1}\!)}\!\! \int\limits_{\pi_{1}=0}^{1} \int\limits_{\pi_{2}=\pi_{1}}^{1}\! \! \!\!P(D_{1} | \pi_{1}\!, \pi_{\!2}) p(\pi_{\!2}) p(\pi_{\!1}) d\pi_{\!2} d\pi_{1} \!,  \end{aligned}  $$

where

4 $$\begin{array}{*{20}l} &P(\pi_{2}>\pi_{1})=\int \limits_{\pi_{1}=0}^{1} \int \limits_{\pi_{2}=\pi_{1}}^{1} p\left(\pi_{1}\right) p\left(\pi_{2} \right) d\pi_{2} d\pi_{1}. \end{array} $$

The unconditional prior distributions *p*(*π*_1_) and *p*(*π*_2_) are defined such that *π*_1_∉ [0,1]⇒*p*(*π*_1_)=0 and *π*_2_∉ [ 0,1]⇒*p*(*π*_2_)=0 (e.g., beta or uniform(0,1) distributions).

Combining (1) and (3) gives the following equation for CEP:

5 $$ \begin{aligned} &{}P(D_{1} |\pi_{2} \!>\! \pi_{1}) \,=\, \frac{1}{P(\pi_{2}\!>\!\pi_{1})}\! \int\limits_{\pi_{1}} \int\limits_{\pi_{2}>\pi_{1}} \, \\[-2pt] &{}\times\!\Phi\!\left[\! \frac{\sqrt{N} \, (\pi_{2}\,-\,\pi_{1}) \,-\, 2 z_{{}_{1-\alpha/2}} \sqrt{\pi(1\,-\,\pi)}}{\sqrt{2 \pi_{2} (1\,-\,\pi_{2}) \,+\, 2 \pi_{1} (1\,-\,\pi_{1})}} \!\right] \!p(\pi_{\!2}) p(\pi_{\!1}) d\pi_{2} d\pi_{\!1}\!.  \end{aligned}  $$

Note, any appropriate sample size and power formulas may be used to evaluate CEP in (5). For example, continuity-corrected versions of (2) or the arcsine approximation [25, 26] may alternatively be utilized instead of (2) to determine sample size, while related power formulas may be used instead of (1) for CEP calculations.

To evaluate CEP under a proposed design, find *N* in (2) for the hypothesized values of *π*_1_ and *π*_2_, significance level *α*, and traditional power level 1−*β*. Numerical integration may then be used to evaluate CEP (5) for the assumed prior distributions *p*(*π*_1_) and *p*(*π*_2_). If CEP for the proposed design is less than 1−*β*, the study is expected to be underpowered under the treatment superiority assumption, and if the CEP is greater than 1−*β*, the study is expected to be overpowered. To ensure that the study is expected to be appropriately powered under the treatment superiority assumption, an iterative search procedure can be used to find the value of the sample size *N* in (5) that gives CEP equal to the threshold of traditional power 1−*β*. The value of *N* that achieves this desired level is denoted *N* ^∗^. As in traditional power, we would like the probability of detecting a difference when a positive difference exists to be high (i.e., 0.80≤1−*β*≤0.90). Pseudo-code 1 outlines the steps for this process.



If the prior distributions put all their mass at a single positive point, essentially becoming a traditional point alternative hypothesis, EP and CEP reduce to the traditional formulation of power. However, for prior distributions where *P*(*π*_1_>*π*_2_)>0, CEP will be greater than EP, with CEP approaching 1 and EP approaching *P*(*π*_2_>*π*_1_) as *N*→*∞*:

$$\begin{aligned} &{}{\text{If }\pi_{2}\!<\!\pi_{1}\!,}\!{\; {\lim}_{{N}\to \infty}\!\Phi\!\! \left[\! \frac{\sqrt{N} \!(\pi_{2}\,-\,\pi_{1}) \,-\, 2 z_{{}_{1-\alpha/2}} \sqrt{\!\pi(1\,-\,\pi)}}{\!\sqrt{2 \pi_{2} (\!1\,-\,\pi_{\!2}) \,+\, 2 \pi_{\!1} (\!1\,-\,\pi_{1}\!)}} \!\right]}{\,=\,\!{\lim}_{z\to -\infty} \!\Phi\!\left[z\right]}{}{=\!0}{} \\ &{}{\text{If}\,\pi_{2}>\!\!\pi_{1}\!,}\!{\; {\lim}_{{N}\to \infty}\!\Phi\!\! \left[ \!\frac{\sqrt{N} (\pi_{2}\,-\,\pi_{1})\! -\! 2 z_{{}_{1-\alpha/2}} \sqrt{\!\pi\!(\!1\,-\,\pi\!)}}{\sqrt{\!2 \pi_{2} (\!1\,-\,\pi_{\!2}) \,+\, 2 \pi_{\!1} \!(\!1\,-\,\pi_{\!1}\!)}} \right]}{={\lim}_{z\to \infty} \Phi\left[z\right]}{}{=1}{} \end{aligned} $$

$$\begin{aligned} &{}{\implies} \!\!\! \!{\lim}_{N \to \infty} \!P(D_{1}\!)\! =\!\! {\lim}_{\!N \!\to\! \infty}\int\limits_{\pi_{1}} \int\limits_{\!\pi_{2}<\pi_{1}} \!\! \!\!\Phi\!\!\left[\! \frac{\!\sqrt{\!N} \! (\pi_{2}\,-\,\pi_{1}) \,-\, 2 z_{{}_{1-\alpha/2}} \!\sqrt{\!\pi(1\,-\,\pi\!)}}{\sqrt{2 \pi_{2} (1\,-\,\pi_{2})\! +\! 2 \pi_{1} \!(1\,-\,\pi_{1})}} \!\right] \!p(\!\pi_{2}\!) p(\pi_{1}\!) \, \!d\pi_{2} \, \!d\pi_{1}\\ &\qquad{+\! {\lim}_{N \to \infty} \int\limits_{\pi_{1}} \int\limits_{\pi_{2} \!>\! \pi_{1}} \!\! \!\Phi\!\!\left[\! \frac{\!\sqrt{N} \! (\pi_{2}\,-\,\pi_{1}\!) \,-\, 2 z_{{}_{1-\alpha/2}} \sqrt{\!\pi\!(1\,-\,\pi\!)}}{\sqrt{2 \pi_{2} (\!1\,-\,\pi_{\!2}\!) \,+\, 2 \pi_{\!1} (1\,-\,\pi_{\!1})}} \right]\! p(\pi_{2}) \, p(\pi_{1}) \, d\pi_{2} \, d\pi_{1}} &\\ &\qquad{= P(\pi_{2}>\pi_{1})} & \end{aligned} $$

When there is no doubt of a beneficial effect (i.e., *P*(*π*_2_>*π*_1_)=1), CEP equals EP.

Previous work in this area almost exclusively uses expected power *P*(*D*_1_) to account for uncertainty in study design parameters [8, 9, 15–24], and finds the sample size that gives the desired level of *P*(*D*_1_). Our preference for using CEP as opposed to EP to inform the design of a study is twofold. EP gives the predictive probability of a positive conclusion, regardless of the truth of the alternative hypothesis. CEP, however, is *conceptually* analogous to traditional power in that it is conditional on the truth of the benefit of the experimental treatment, which provides a more familiar framework for setting the desired level of CEP for a study. Secondly, if *P*(*π*_1_>*π*_2_)>0, then EP will not approach 1 as the sample size goes to infinity because ${\lim }_{N\to \infty } P(D_{1})=1-P(\pi _{1}>\pi _{2})$. CEP, however, is conditioned on *π*_2_>*π*_1_, so it approaches 1 as the sample size increases since ${\lim }_{N\to \infty } P(D_{1} \,|\, \pi _{2} > \pi _{1}) = \frac {1-P(\pi _{1}>\pi _{2})}{P(\pi _{2}>\pi _{1})}=1$. Thus, CEP is also more *mathematically* analogous to traditional power in that the probability of correctly reaching a positive conclusion is assured as the sample size goes to infinity.

### Prior distributions

The prior distributions *p*(*π*_1_) and *p*(*π*_2_) reflect the current knowledge about the response rate in each treatment group before the trial is conducted. In the design phase of a clinical trial, a review of the literature is often performed. This collection of prior evidence forms a natural foundation for specifying the prior distributions. Historical data are commonly pooled using traditional meta-analysis techniques to calculate an overall point estimate [27, 28]; however, a Bayesian random-effects meta-analysis [29–31] may be more appropriate when the goal is to hypothesize a prior distribution. The priors can also incorporate the pre-trial consensus of experts in the field [9] or Phase II trial data [22]. Translating and combining prior evidence and opinions to form a prior distribution is often hailed as the most challenging part of using a Bayesian framework [7], and several works [32–35] describe techniques for eliciting a prior distribution.

A beta distribution, which is defined on the interval [ 0,1], can be used to describe initial beliefs about the parameters *π*_1_ and *π*_2_. If *π*_*j*_∼*Beta*(*a,b*), then

$$ p(\pi_{j}) = \frac{\Gamma(a+b)}{\Gamma(a) \, \Gamma(b)} \pi_{j}^{a-1} \, (1-\pi_{j})^{b-1} $$

where shape parameters *a*>0 and *b*>0. The mean, variance, and mode of the prior distribution are given by: *μ*=*a*/(*a*+*b*),*τ* ^2^=*ab*/((*a*+*b*)^2^(*a*+*b*+1)), and *m*=(*a*−1)/(*a*+*b*−2) for *a,b*>1, respectively. For fixed *μ*, larger values of *a* and *b* decrease *τ* ^2^. One may choose the shape parameters *a* and *b* by fixing the mean and variance of the distribution at fixed values *μ* and *τ* ^2^, which yields *a*=*μ* ^2^(1−*μ*)/*τ* ^2^−*μ* and *b*=*a*(1−*μ*)/*μ*. For skewed distributions, one may wish to describe central tendency using the mode *m* rather than the mean. Under a traditional design, the difference in modes, *m*_2_−*m*_1_, is a natural estimate for the hypothesized difference in proportions. When fixing *m* and *τ* ^2^, the corresponding value of *b* may be found by solving the general cubic equation *Ab* ^3^+*Bb* ^2^+*Cb*+*D*=0, with coefficients

$$\begin{aligned} &{}A=-\frac{m^{3}}{(m-1)^{3}}+\frac{3m^{2}}{(m-1)^{2}}-\frac{3m}{(m-1)}+1\\ &{}B=\frac{6m^{3}-3m^{2}}{(m-1)^{3}}+\frac{-11m^{2}+6m}{(m-1)^{2}}+\frac{4m+\frac{m}{\tau^{2}}-3}{(m-1)}+1\\ &{}C=\frac{-12m^{3}+12m^{2}-3m}{(m-1)^{3}}+\frac{8m^{2}-10m+3}{(m-1)^{2}}-\frac{4m-2+\frac{1-2m}{\tau^{2}}}{(m-1)}\\ &{}D=\frac{8m^{3}-12m^{2}+6m-1}{(m-1)^{3}}+\frac{4m^{2}-4m+1}{(m-1)^{2}}. \end{aligned} $$

The corresponding value of *a* is given by $a=\frac {2m-mb-1}{m-1}$. (Table 2 in the Appendix reports the values of *a* and *b* for given *m* and *τ* ^2^.) Notice that for a given variance *τ* ^2^, the value of *a* when the mode =*m* equals the value of *b* when the mode =1−*m*. Thus, when *m*=0.5,*a*=*b*.

A uniform prior distribution may also be assumed for *π*_*j*_ with limits within the interval [ 0,1]. The uniform prior has lower bound *a* and upper bound *b*, or *π*_*j*_∼U(*a,b*), and is constant over the range [ *a,b*]. The prior is centered at *μ*=(*a*+*b*)/2 with variance *τ* ^2^=(*b*−*a*)^2^/12. The non-informative prior distribution that assumes no values of *π*_*j*_ are more probable than any others is U(0,1)≡*Beta*(1,1). One may also restrict the range of the uniform distribution to focus on smaller ranges for *π*_1_ and *π*_2_. Rather than setting the lower and upper bounds of the uniform, one may set the mean *μ*<1 and variance $\tau ^{2} < \frac {\min (\mu ^{2}, (1-\mu)^{2})}{3}$ of the prior distribution, which gives lower bound $a = \mu - \sqrt {3 \, \tau ^{2}} $ and upper bound $b = \mu + \sqrt {3 \, \tau ^{2}}$. Again, under a traditional design, the difference in means *μ*_2_−*μ*_1_ is a natural estimate for the hypothesized difference in proportions when presented with uniform prior evidence. (Table 3 in the Appendix reports the values of *a* and *b* for given *μ* and *τ* ^2^.) Notice that restrictions exist for the variances assumed for certain means to maintain bounds between [ 0,1].

## Results

The procedures described in the “Methods” Section were applied to a set of notional scenarios to compare traditionally designed studies to those designed using CEP. The integration step of Pseudo-code 1 was approximated using Riemann sums with step size 0.0001.

**Fig. 2:**
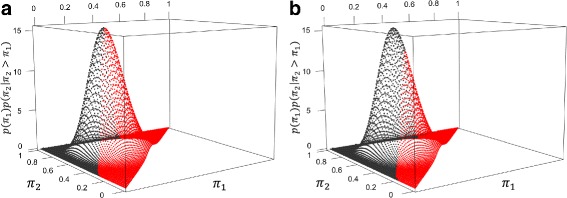
Conditional joint prior density *p*(*π*_1_)*p*(*π*_2_|*π*_2_>*π*_1_) for *π*_1_∼*Beta*(6.62,14.11) and *π*_2_∼*Beta*(14.11,6.62). **a** Highlighting region where power <80*%* under a traditional design. **b** Highlighting region where power <80*%* under a CEP design

An example scenario assumed beta-distributed priors for *π*_1_ and *π*_2_, such that *π*_1_∼*Beta*(6.62,14.11) and *π*_2_∼*Beta*(14.11,6.62). For this scenario, a traditionally designed study would select a sample size of *N*=48 based on (2) to achieve 80*%* power and a two-sided type I error of 5*%*, with hypothesized values of *π*_1_=mode(*Beta*(6.62,14.11))=0.3 and *π*_2_=mode(*Beta*(14.11,6,62))=0.7. However, based on the assumed prior distributions, a study with a sample size of 48 could achieve less than 80*%* power when *π*_1_≠0.3 or *π*_2_≠0.7. In fact, based on (5), the study with sample size *N*=48 would give CEP=67.8*%*. Figure 2 a displays the joint distribution of *π*_1_ and *π*_2_, conditional on *π*_2_>*π*_1_, and highlights the region where power would be less than 80*%* under a traditional design when the sample size is *N*=48. For this scenario, the study with sample size *N*=48 would achieve power less than the target value in more than 56*%* of instances when *π*_2_>*π*_1_.

For the same scenario, a CEP-designed study would select a sample size of *N* ^∗^=80 based on Pseudo-code 1 to achieve 80*%* CEP with a two-sided type I error of 5*%*. Figure 2 b displays the joint distribution of *π*_1_ and *π*_2_, conditional on *π*_2_>*π*_1_, and highlights the region where power would be less than 80*%* under a CEP design when the sample size is *N* ^∗^=80. For this scenario, the study with sample size *N* ^∗^=80 would achieve power less than the target value in approximately 33*%* of instances when *π*_2_>*π*_1_. Note that the intersection of the two regions corresponds to values of *π*_1_ and *π*_2_ that give power from (1) equal to 80*%* with sample size *N*=80.

The probability of achieving power at least equal to the target value, conditional on the experimental treatment’s superiority (*π*_2_>*π*_1_), is here termed the *performance* of the design. While CEP provides a point estimate of power under the treatment superiority assumption, performance indicates how robust the design is. The performance of the design is given by:

6 $$ \begin{aligned} &{}\text{Performance}=\! \frac{1}{P(\!\pi_{2}\!\!>\!\!\pi_{1}\!)} \!\int\limits_{\pi_{1}} \int\limits_{\!\pi_{2}>\pi_{1}} \!\!\! \textsf{\!F}\!\left(N\!,\!\pi_{\!1}\!,\pi_{\!2}\!,z_{{}_{1-\alpha/2}}\!\right) \!p(\pi_{\!2}) \, p(\pi_{\!1}) d\pi_{2} d\pi_{1}\!, \end{aligned}  $$

where

$$\begin{aligned} &{}\textsf{F}\left(N\!,\!\pi_{1\!},\pi_{2}\!,z_{{}_{\!1-\alpha/2}}\right)\,=\, \begin{cases} \!1 & \text{\!\!\!if }\!\Phi\!\left[\! \!\frac{\sqrt{N} \, \!(\pi_{2}\,-\,\pi_{1}) \,-\, 2 z_{{}_{1-\alpha/2}} \sqrt{\!\pi(1\!\,-\,\pi)}}{\!\sqrt{\!2 \pi_{2} (1\,-\,\pi_{2}) \,+\, 2 \pi_{1} (1\,-\,\pi_{1}\!)}}\! \right]\! \!\geq\!\! 1\,-\,\beta\!,\\ \!0 & \text{\!\!\!otherwise} \end{cases} \end{aligned} $$

Thus, the traditionally designed study from the example scenario produced a performance of (100−56)*%*=44*%*, while the CEP design, which explicitly accounts for uncertainty, produced a more robust performance of (100−33)*%*=67*%*. However, this increase in performance required an increase in sample size from *N*=48 to *N* ^∗^=80. The increase in performance divided by the increase in sample size is here termed the *marginal benefit* for the scenario due to CEP. The marginal benefit for the example scenario due to CEP is given by (67−44)*%*/(80−48)=0.71*%*. If there is no uncertainty in the design parameters, then there would be no marginal benefit due to CEP, since the probability of achieving less than the target power would be assumed 0 for a traditionally designed study and the CEP-designed study would give *N* ^∗^=*N*. On the other hand, if the uncertainty in the design parameters is very large, the marginal benefit may approach 0, since the CEP-designed study could give *N* ^∗^>>*N* with limited increase in performance. This is important to consider, since a very small marginal benefit could make it impractical to achieve a desired value for CEP or a desired threshold of performance.

Since the performance and marginal benefit result from the prior distributions of *π*_1_ and *π*_2_, several notional scenarios were evaluated to explore the relationship between prior distributions, CEP, and performance. Tables 4, 5 and 6 in the Appendix display the results of several scenarios that assumed Beta-distributed priors for *π*_1_ and *π*_2_. The mode and variance of *p*(*π*_*j*_),*j*=1,2, are denoted *m*_*j*_ and $\tau ^{2}_{j}$, respectively. The procedure for generating the results from Table 4 in the Appendix, for which $\tau ^{2}_{1}=\tau ^{2}_{2}$, is given below:

1. The modes, *m*_1_ and *m*_2_, and variances, $\tau ^{2}_{1}=\tau ^{2}_{2}$, were used to hypothesize a beta prior distribution for *π*_1_ and *π*_2_, respectively.
2. For each pair of prior distributions (*p*(*π*_1_),*p*(*π*_2_)) considered:
  - Traditional sample size is found using (2) by setting the hypothesized values of *π*_1_ and *π*_2_ equal to the mode of each prior, *m*_1_ and *m*_2_, respectively. Two-sided type I error *α*=0.05 and traditional power 1−*β*=0.80 are assumed. Traditional sample size is denoted $\hat {N}$. If $\hat {N}$ is odd, the sample size is increased by 1 to provide equal sample size for both groups.
  - The CEP of the traditional design is found using (5), with $N=\hat {N}$, two-sided *α*=0.05, and 1−*β*=0.80.
  - The performance of the traditional design is found using (6), with $N=\hat {N}$, two-sided *α*=0.05, and 1−*β*=0.80.
  - The smallest sample size for which CEP evaluates to ≥1−*β* is found using Pseudo-Code 1 and is denoted *N* ^∗^. If *N* ^∗^ is odd, the sample size is increased by 1 to provide equal sample size for both groups.
  - The probability of a positive treatment effect, *P*(*π*_2_>*π*_1_), is found using (4) with Riemann sum integral approximations.
  - The conditional expected difference, *E*(*π*_2_−*π*_1_|*π*_2_>*π*_1_), is found using Riemann sum integral approximations of
    $$\begin{aligned} {}E(\pi_{2} \!- \!\pi_{1}|\pi_{2} \!\!>\!\! \pi_{1})\,=\,\frac{1}{P(\pi_{2}\!\!>\!\!\pi_{1})}\!\! \int \limits_{\pi_{1}=0}^{1} \int \limits_{\pi_{2}=\pi_{1}}^{1} \!\!(\pi_{2}\,-\,\pi_{1}) p\!\left(\pi_{1}\right) \!p\!\left(\pi_{2} \right) \!d\pi_{2} d\pi_{1}. \end{aligned} $$
  - The performance of the CEP design is found using (6), with *N*=*N* ^∗^, two-sided *α*=0.05, and 1−*β*=0.80.
  - The marginal benefit due to CEP for the scenario is found by dividing the difference between the CEP design performance and the traditional design performance by the difference between the CEP sample size and the traditional sample size, $N^{*}-\hat {N}$.

Table 4 in the Appendix shows that when *m*_2_−*m*_1_>1/3, the performance of the traditional design decreases as $\tau _{1}^{2}=\tau _{2}^{2}$ increases. This is explained by the fact that the conditional expected difference is less than the hypothesized difference that was used in the traditional design sample size calculation. This occurs for *m*_2_−*m*_1_>1/3 since both prior distributions are approaching U(0,1) as $\tau _{1}^{2}=\tau _{2}^{2}$ increases, and *E*(*π*_2_−*π*_1_|*π*_2_>*π*_1_)=1/3 for *π*_1_,*π*_2_∼U(0,1). Thus, when *m*_2_−*m*_1_<1/3, the performance of the traditional design *increases* as $\tau _{1}^{2}=\tau _{2}^{2}$ increases since the hypothesized difference is *less* than the limit of the conditional expected difference. When *m*_2_−*m*_1_ is smaller than *E*(*π*_2_−*π*_1_|*π*_2_>*π*_1_), CEP will be high for a traditional design with hypothesized difference *m*_2_−*m*_1_, since it is designed to detect a difference smaller than the expected difference.

The procedure was also applied to scenarios where $\tau _{1}^{2} = 0.001$ and $\tau _{2}^{2} > 0.001$ (Table 5 in the Appendix) and scenarios where $\tau _{1}^{2} = 0.08$ and $\tau _{2}^{2} < 0.08$ (Table 6 in the Appendix), corresponding to small and large uncertainty, respectively, in the proportion experiencing the outcome in the control group. Table 5 in the Appendix shows that the performance of the traditional design is similar to the performance seen in Table 4 in the Appendix. However, when $\tau _{1}^{2}$ is fixed at 0.001,*E*(*π*_2_−*π*_1_|*π*_2_>*π*_1_) begins near *m*_2_−*m*_1_ and approaches (1−*m*_1_)/2 as $\tau _{2}^{2}$ increases because *p*(*π*_2_|*π*_2_>*π*_1_) is approaching U(*m*_1_,1). Thus, when *m*_2_−*m*_1_>(1−*m*_1_)/2, the performance of the traditional design decreases as $\tau _{2}^{2}$ increases, and when *m*_2_−*m*_1_<(1−*m*_1_)/2, the performance of the traditional design increases as $\tau _{2}^{2}$ increases.

When $\tau _{1}^{2}$ is fixed at 0.08,*E*(*π*_2_−*π*_1_|*π*_2_>*π*_1_) approaches 1/3 from *m*_2_/2. If *E*(*π*_2_−*π*_1_|*π*_2_>*π*_1_) is increasing towards 1/3 as $\tau _{2}^{2}$ increases, then the performance of the traditional design will increase. If *E*(*π*_2_−*π*_1_|*π*_2_>*π*_1_) decreases towards 1/3 as $\tau _{2}^{2}$ increases, then the performance of the traditional design will decrease. If *m*_2_/2>1/3, then the performance of the traditional design will decrease as $\tau _{2}^{2}$ increases. This happens because, as $\tau _{2}^{2}$ increases, the hypothesized difference is decreasing from *m*_2_/2 to 1/3. The behavior of the traditional design is summarized in Table 1.

**Table 1:** Analysis of traditional design performance

Uncertainty	*m*_2_−*m*_1_	Performance
$\tau _{1}^{2}=\tau _{2}^{2}$	<1/3	Increases as $\tau _{1}^{2}=\tau _{2}^{2}$ increases
	>1/3	Decreases as $\tau _{1}^{2}=\tau _{2}^{2}$ increases
$\tau _{1}^{2}$ small	<(1−*m*_1_)/2	Increases as $\tau _{2}^{2}$ increases
	>(1−*m*_1_)/2	Decreases as $\tau _{2}^{2}$ increases
$\tau _{1}^{2}$ large	>2/3−*m*_1_	Increases as $\tau _{2}^{2}$ increases
	<2/3−*m*_1_	Decreases as $\tau _{2}^{2}$ increases

Excursions with uniform priors were performed. Table 7 in the Appendix shows that the performance of a traditional design under a uniform prior is similar to the performance observed in Table 4 in the Appendix. However, fewer trends are visible because the parameters of the uniform distribution are more restricted than the parameters of the beta distribution.

As expected, the performance of the CEP design changes minimally as $\tau _{1}^{2}=\tau _{2}^{2}$ increases, since *N* ^∗^ is chosen to explicitly account for changes in $\tau _{1}^{2}=\tau _{2}^{2}$. Note, *N* ^∗^ is directly tied to *E*(*π*_2_−*π*_1_|*π*_2_>*π*_1_): *N* ^∗^ increases as the conditional expected difference decreases, and *N* ^∗^ decreases as the conditional expected difference increases. This occurs because increasing the variability can increase the conditional expected difference if the resulting conditional priors give more relative weight to larger differences and less relative weight to smaller differences compared to the unconditional priors. This is more likely to occur when *m*_1_ is large, since increasing the variability when *m*_1_ is large will make smaller values of *π*_1_ more likely due to the condition that *π*_2_>*π*_1_. Similarly, when *m*_2_ is small, larger values of *π*_2_ are more likely under the assumption that *π*_2_>*π*_1_.

The marginal benefit due to CEP is greatest for small values of $\tau _{1}^{2}=\tau _{2}^{2}$. This is so because the relative difference between $\hat {N}$ and *N* ^∗^ is smallest when the uncertainty is low (i.e., when the traditional assumptions closely approximate the CEP assumptions). However, the marginal benefit due to CEP decreases minimally or remains constant as the uncertainty increases because the difference in performance is always less than 1, while the difference in sample size, $N^{*}-\hat {N}$, can be greater than 200 in some cases. Furthermore, as $\tau _{1}^{2}=\tau _{2}^{2}$ increases, the performance of the traditional design can improve even though $\hat {N}$ remains constant, while *N* ^∗^ may have to increase to maintain the performance of the CEP design.

When $\tau _{1}^{2}$ is fixed at 0.001, the performance of the CEP design remains stable at approximately 0.7. However, the marginal benefit is greater with fixed, low uncertainty in *π*_1_ compared with the changing uncertainty in Table 4 in the Appendix. The sample size required to achieve CEP of 1−*β* with fixed $\tau _{1}^{2}$ is reduced compared to scenarios with changing $\tau _{1}^{2}$. This is because uncertainty in the control group is small, which indicates that reducing the uncertainty in the control parameter can increase the benefit of CEP to the study.

When $\tau _{1}^{2}$ is fixed at 0.08, the performance of the CEP design remains stable at approximately 0.71. However, the marginal benefit is very small because *N* ^∗^ is always greater than that in Table 4 or Table 5 in the Appendix due to the larger uncertainty in *π*_1_. Again, this demonstrates that it is beneficial to minimize the uncertainty in *π*_1_ to increase the marginal benefit.

Note that for small differences in *m*_2_−*m*_1_ and any large variance, the CEP design can reduce the sample size from the value determined from a traditional design. The reason is that increased uncertainty under the treatment superiority assumption increases the likelihood of differences greater than *m*_2_−*m*_1_.

## Discussion

Many underpowered clinical trials are conducted with limited justification for the chosen study parameters used to determine the required sample size [36, 37] with scientific, economic, and ethical implications [36, 38]. While sample size calculations based on traditional power assume no uncertainty in the study parameters, the hybrid classical and Bayesian procedure presented here formally accounts for the uncertainty in the study parameters by incorporating the prior distributions for *π*_1_ and *π*_2_ into the calculation of conditional expected power (CEP). This method allows available evidence on both the magnitude and the variability surrounding the parameters to play a formal role in determining study power and sample size.

In this paper, we explored several notional scenarios to compare the performance of the CEP design to that of a design based on traditional power. We show that if there is uncertainty in the study parameters and a distribution of plausible values for *π*_1_ and *π*_2_, the performance of the CEP design is more consistent and robust than that of traditional designs based on point estimates for the study parameters. Traditional sample size calculations based on point estimates for the hypothesized study parameters tend to underestimate the required sample size needed to account for the uncertainty in the parameters.

The scenarios demonstrate that reducing uncertainty in the control parameter *π*_1_ can lead to greater benefit from the CEP-designed study, because the relative difference between $\hat {N}$ and *N* ^∗^ is smallest when uncertainty is low. Therefore, it is worthwhile to use historical information to reduce the variability in the control group proportion rather than focusing only on the prior for the experimental treatment group. Nonetheless, when there is significant overlap between the prior distributions and a small hypothesized difference *m*_2_−*m*_1_, traditional study designs can be overpowered under the treatment superiority assumption compared to the CEP design, and the CEP design would result in a smaller sample size. This happens because increased uncertainty under the treatment superiority assumption increases the relative likelihood of differences greater than *m*_2_−*m*_1_.

In the scenarios we evaluated, the performance of the traditional design was highly dependent on the prior distributions but exhibited predictable behavior. The CEP design, however, consistently generated performance near 70*%* across all scenarios. This indicates that power greater than the target 1−*β* would not be uncommon for a CEP design. This begs the question of whether or not 1−*β* is an appropriate target for CEP, since it could apparently lead to overpowered studies. To avoid this issue, one may use a lower target for CEP or instead design the study using a target value of performance and use our iterative *N* ^∗^ search to find the design that achieves acceptable performance.

Additionally, when comparing the method based on CEP to similar methods based on expected power, the sample size from a CEP design will always be less than or equal to the sample size required to achieve equivalent EP. While pure Bayesian methods of sample size determination that compute prior effective sample size to count the information contained in the prior towards the current study will generally yield a smaller sample size than traditional frequentist methods [10], the method presented here does not assume that prior information will be incorporated into the final analysis.

## Conclusions

The hybrid classical and Bayesian procedure presented here integrates available prior information about the study design parameters into the calculation of study sample size for a binary endpoint. This method allows prior information on both the magnitude and uncertainty surrounding the parameters *π*_1_ and *π*_2_ to inform the design of the current study through the use of conditional expected power. When there is a distribution of plausible study parameters, the design based on conditional expected power tends to outperform the traditional design. Note that if the determined sample size *N* ^∗^ is greater than what can be feasibly recruited in the proposed trial, this may indicate excessive uncertainty about the study parameters and should encourage serious discussion concerning the advisability of the study. Thus, we do not recommend that *N* ^∗^ be blindly used as the final study sample size, but we hope that this method encourages a careful synthesis of the prior information and motivates thoughtful discussion about the feasibility of the study in order to reduce the number of poorly designed, underpowered studies that are conducted.

## Appendix

Table 2 presents the values of shape paramaters [a, b] for given *m* and *τ* ^2^ for the beta distribution. Table 3 reports the values of minimum and maximum parameters [ *a,b*] for given *μ* and *τ* ^2^ for the uniform distribution.

**Table 2:** Shape parameters [ *a,b*] of beta distribution given mode *m* and variance *τ* ^2^

*m*/ *τ* ^2^	0.001	0.005	0.01	0.015	0.02	0.025	0.03	0.035	0.04	0.045	0.05	0.055	0.06	0.065	0.07	0.075	0.08
0.01	[1.33,33.99]	[1.12,12.73]	[1.07,8.18]	[1.05,6.21]	[1.04,5.05]	[1.03,4.26]	[1.03,3.67]	[1.02,3.21]	[1.02,2.84]	[1.02,2.53]	[1.01,2.26]	[1.01,2.03]	[1.01,1.82]	[1.01,1.63]	[1.01,1.45]	[1,1.28]	[1,1.12]
0.05	[3.89,55.92]	[1.77,15.55]	[1.43,9.21]	[1.3,6.74]	[1.23,5.36]	[1.18,4.45]	[1.15,3.79]	[1.12,3.29]	[1.1,2.89]	[1.08,2.56]	[1.07,2.28]	[1.05,2.03]	[1.04,1.82]	[1.03,1.62]	[1.02,1.44]	[1.02,1.28]	[1.01,1.11]
0.1	[10.36,85.26]	[3.05,19.4]	[2.07,10.59]	[1.72,7.44]	[1.53,5.76]	[1.41,4.7]	[1.33,3.95]	[1.27,3.39]	[1.22,2.95]	[1.18,2.59]	[1.14,2.3]	[1.12,2.04]	[1.09,1.82]	[1.07,1.62]	[1.05,1.44]	[1.03,1.27]	[1.01,1.11]
0.15	[20.24,110]	[4.88,23.01]	[2.93,11.91]	[2.25,8.1]	[1.91,6.14]	[1.69,4.92]	[1.55,4.09]	[1.44,3.48]	[1.35,3]	[1.29,2.62]	[1.23,2.31]	[1.18,2.04]	[1.14,1.81]	[1.11,1.61]	[1.08,1.43]	[1.05,1.26]	[1.02,1.1]
0.2	[32.84,128.38]	[7.22,25.89]	[4,13]	[2.91,8.65]	[2.36,6.45]	[2.03,5.11]	[1.8,4.2]	[1.64,3.54]	[1.51,3.04]	[1.41,2.64]	[1.33,2.31]	[1.26,2.04]	[1.2,1.8]	[1.15,1.6]	[1.1,1.41]	[1.06,1.25]	[1.02,1.1]
0.25	[47.46,140.37]	[9.95,27.84]	[5.25,13.75]	[3.68,9.03]	[2.89,6.66]	[2.41,5.23]	[2.09,4.27]	[1.86,3.57]	[1.68,3.05]	[1.55,2.63]	[1.43,2.3]	[1.34,2.02]	[1.26,1.78]	[1.19,1.58]	[1.13,1.4]	[1.08,1.24]	[1.03,1.09]
0.3	[63.33,146.43]	[12.92,28.82]	[6.62,14.11]	[4.52,9.21]	[3.46,6.75]	[2.83,5.27]	[2.4,4.28]	[2.1,3.57]	[1.87,3.03]	[1.69,2.61]	[1.55,2.27]	[1.43,1.99]	[1.33,1.76]	[1.24,1.56]	[1.16,1.38]	[1.1,1.23]	[1.04,1.09]
0.35	[79.71,147.18]	[16.01,28.88]	[8.05,14.09]	[5.39,9.16]	[4.06,6.69]	[3.27,5.21]	[2.73,4.22]	[2.35,3.51]	[2.07,2.98]	[1.84,2.56]	[1.66,2.23]	[1.52,1.96]	[1.39,1.73]	[1.29,1.53]	[1.2,1.36]	[1.12,1.21]	[1.04,1.08]
0.4	[95.87,143.3]	[19.07,28.1]	[9.47,13.7]	[6.27,8.9]	[4.67,6.5]	[3.71,5.06]	[3.06,4.1]	[2.61,3.41]	[2.26,2.89]	[2,2.49]	[1.78,2.17]	[1.61,1.91]	[1.46,1.69]	[1.33,1.5]	[1.23,1.34]	[1.13,1.2]	[1.05,1.08]
0.45	[111.04,135.5]	[21.94,26.6]	[10.81,12.99]	[7.09,8.45]	[5.24,6.18]	[4.12,4.82]	[3.38,3.91]	[2.85,3.26]	[2.45,2.77]	[2.14,2.4]	[1.89,2.09]	[1.69,1.85]	[1.52,1.64]	[1.38,1.46]	[1.26,1.31]	[1.15,1.18]	[1.06,1.07]
0.5	[124.5,124.5]	[24.5,24.5]	[12,12]	[7.83,7.83]	[5.75,5.75]	[4.5,4.5]	[3.67,3.67]	[3.07,3.07]	[2.63,2.63]	[2.28,2.28]	[2,2]	[1.77,1.77]	[1.58,1.58]	[1.42,1.42]	[1.29,1.29]	[1.17,1.17]	[1.06,1.06]
0.55	[135.5,111.04]	[26.6,21.94]	[12.99,10.81]	[8.45,7.09]	[6.18,5.24]	[4.82,4.12]	[3.91,3.38]	[3.26,2.85]	[2.77,2.45]	[2.4,2.14]	[2.09,1.89]	[1.85,1.69]	[1.64,1.52]	[1.46,1.38]	[1.31,1.26]	[1.18,1.15]	[1.07,1.06]
0.6	[143.3,95.87]	[28.1,19.07]	[13.7,9.47]	[8.9,6.27]	[6.5,4.67]	[5.06,3.71]	[4.1,3.06]	[3.41,2.61]	[2.89,2.26]	[2.49,2]	[2.17,1.78]	[1.91,1.61]	[1.69,1.46]	[1.5,1.33]	[1.34,1.23]	[1.2,1.13]	[1.08,1.05]
0.65	[147.18,79.71]	[28.88,16.01]	[14.09,8.05]	[9.16,5.39]	[6.69,4.06]	[5.21,3.27]	[4.22,2.73]	[3.51,2.35]	[2.98,2.07]	[2.56,1.84]	[2.23,1.66]	[1.96,1.52]	[1.73,1.39]	[1.53,1.29]	[1.36,1.2]	[1.21,1.12]	[1.08,1.04]
0.7	[146.43,63.33]	[28.82,12.92]	[14.11,6.62]	[9.21,4.52]	[6.75,3.46]	[5.27,2.83]	[4.28,2.4]	[3.57,2.1]	[3.03,1.87]	[2.61,1.69]	[2.27,1.55]	[1.99,1.43]	[1.76,1.33]	[1.56,1.24]	[1.38,1.16]	[1.23,1.1]	[1.09,1.04]
0.75	[140.37,47.46]	[27.84,9.95]	[13.75,5.25]	[9.03,3.68]	[6.66,2.89]	[5.23,2.41]	[4.27,2.09]	[3.57,1.86]	[3.05,1.68]	[2.63,1.55]	[2.3,1.43]	[2.02,1.34]	[1.78,1.26]	[1.58,1.19]	[1.4,1.13]	[1.24,1.08]	[1.09,1.03]
0.8	[128.38,32.84]	[25.89,7.22]	[13,4]	[8.65,2.91]	[6.45,2.36]	[5.11,2.03]	[4.2,1.8]	[3.54,1.64]	[3.04,1.51]	[2.64,1.41]	[2.31,1.33]	[2.04,1.26]	[1.8,1.2]	[1.6,1.15]	[1.41,1.1]	[1.25,1.06]	[1.1,1.02]
0.85	[110,20.24]	[23.01,4.88]	[11.91,2.93]	[8.1,2.25]	[6.14,1.91]	[4.92,1.69]	[4.09,1.55]	[3.48,1.44]	[3,1.35]	[2.62,1.29]	[2.31,1.23]	[2.04,1.18]	[1.81,1.14]	[1.61,1.11]	[1.43,1.08]	[1.26,1.05]	[1.1,1.02]
0.9	[85.26,10.36]	[19.4,3.05]	[10.59,2.07]	[7.44,1.72]	[5.76,1.53]	[4.7,1.41]	[3.95,1.33]	[3.39,1.27]	[2.95,1.22]	[2.59,1.18]	[2.3,1.14]	[2.04,1.12]	[1.82,1.09]	[1.62,1.07]	[1.44,1.05]	[1.27,1.03]	[1.11,1.01]
0.95	[55.93,3.89]	[15.55,1.77]	[9.21,1.43]	[6.74,1.3]	[5.36,1.23]	[4.45,1.18]	[3.79,1.15]	[3.29,1.12]	[2.89,1.1]	[2.56,1.08]	[2.28,1.07]	[2.03,1.05]	[1.82,1.04]	[1.62,1.03]	[1.44,1.02]	[1.28,1.02]	[1.11,1.01]
0.99	[33.99,1.33]	[12.73,1.12]	[8.18,1.07]	[6.21,1.05]	[5.05,1.04]	[4.26,1.03]	[3.67,1.03]	[3.21,1.02]	[2.84,1.02]	[2.53,1.02]	[2.26,1.01]	[2.03,1.01]	[1.82,1.01]	[1.63,1.01]	[1.45,1.01]	[1.28,1.003]	[1.12,1.001]

**Table 3:** Minimum and maximum parameters [ *a,b*] of uniform distribution given mean *μ* and variance *τ* ^2^

*μ*/ *τ* ^2^	0.001	0.005	0.01	0.015	0.02	0.025	0.03	0.035	0.04	0.045	0.05	0.055	0.06	0.065	0.07	0.075	0.08	0.0833
0.01																		
0.05																		
0.1	[0.045,0.155]																	
0.15	[0.095,0.205]	[0.028,0.272]																
0.2	[0.145,0.255]	[0.078,0.322]	[0.027,0.373]															
0.25	[0.195,0.305]	[0.128,0.372]	[0.077,0.423]	[0.038,0.462]	[0.005,0.495]													
0.3	[0.245,0.355]	[0.178,0.422]	[0.127,0.473]	[0.088,0.512]	[0.055,0.545]	[0.026,0.574]	[0,0.6]											
0.35	[0.295,0.405]	[0.228,0.472]	[0.177,0.523]	[0.138,0.562]	[0.105,0.595]	[0.076,0.624]	[0.05,0.65]	[0.026,0.674]	[0.004,0.696]									
0.4	[0.345,0.455]	[0.278,0.522]	[0.227,0.573]	[0.188,0.612]	[0.155,0.645]	[0.126,0.674]	[0.1,0.7]	[0.076,0.724]	[0.054,0.746]	[0.033,0.767]	[0.013,0.787]							
0.45	[0.395,0.505]	[0.328,0.572]	[0.277,0.623]	[0.238,0.662]	[0.205,0.695]	[0.176,0.724]	[0.15,0.75]	[0.126,0.774]	[0.104,0.796]	[0.083,0.817]	[0.063,0.837]	[0.044,0.856]	[0.026,0.874]	[0.008,0.892]				
0.5	[0.445,0.555]	[0.378,0.622]	[0.327,0.673]	[0.288,0.712]	[0.255,0.745]	[0.226,0.774]	[0.2,0.8]	[0.176,0.824]	[0.154,0.846]	[0.133,0.867]	[0.113,0.887]	[0.094,0.906]	[0.076,0.924]	[0.058,0.942]	[0.042,0.958]	[0.026,0.974]	[0.01,0.99]	[0,1]
0.55	[0.495,0.605]	[0.428,0.672]	[0.377,0.723]	[0.338,0.762]	[0.305,0.795]	[0.276,0.824]	[0.25,0.85]	[0.226,0.874]	[0.204,0.896]	[0.183,0.917]	[0.163,0.937]	[0.144,0.956]	[0.126,0.974]	[0.108,0.992]				
0.6	[0.545,0.655]	[0.478,0.722]	[0.427,0.773]	[0.388,0.812]	[0.355,0.845]	[0.326,0.874]	[0.3,0.9]	[0.276,0.924]	[0.254,0.946]	[0.233,0.967]	[0.213,0.987]							
0.65	[0.595,0.705]	[0.528,0.772]	[0.477,0.823]	[0.438,0.862]	[0.405,0.895]	[0.376,0.924]	[0.35,0.95]	[0.326,0.974]	[0.304,0.996]									
0.7	[0.645,0.755]	[0.578,0.822]	[0.527,0.873]	[0.488,0.912]	[0.455,0.945]	[0.426,0.974]	[0.4,1]											
0.75	[0.695,0.805]	[0.628,0.872]	[0.577,0.923]	[0.538,0.962]	[0.505,0.995]													
0.8	[0.745,0.855]	[0.678,0.922]	[0.627,0.973]															
0.85	[0.795,0.905]	[0.728,0.972]																
0.9	[0.845,0.955]																	
0.95																		
0.99																		

**Table 4:** Sample scenarios assuming beta priors *p*(*π*_1_) and *p*(*π*_2_) where $\tau _{1}^{2}=\tau _{2}^{2}$. Hypothesized values of *π*_1_ and *π*_2_ set equal to *m*_1_ and *m*_2_, respectively, under the traditional design. Two-sided *α*=0.05 and 1−*β*=0.80 assumed

		Traditional design			CEP design				
(*m*_1_,*m*_2_)	$\tau _{1}^{2}=\tau _{2}^{2}$	$\hat {N}$	Performance	CEP	*N* ^∗^	Performance	*E*(*π*_2_−*π*_1_|*π*_2_>*π*_1_)	*P*(*π*_2_>*π*_1_)	Marginal benefit
(0.1,0.9)	0.001	10	0.797	0.518	12	0.742	0.783	1	0.1120
	0.01	10	0.622	0.242	16	0.627	0.674	1	0.0642
	0.02	10	0.508	0.164	26	0.673	0.585	0.993	0.0318
	0.03	10	0.433	0.123	40	0.687	0.519	0.965	0.0188
	0.04	10	0.381	0.099	60	0.698	0.469	0.915	0.0120
	0.05	10	0.342	0.082	86	0.705	0.429	0.846	0.0082
	0.06	10	0.311	0.070	120	0.710	0.397	0.761	0.0058
	0.07	10	0.285	0.060	160	0.713	0.369	0.662	0.0044
	0.08	10	0.262	0.052	214	0.716	0.342	0.545	0.0033
(0.1,0.8) or (0.2,0.9)	0.001	14	0.804	0.559	14	0.559	0.688	1	0
	0.01	14	0.656	0.314	22	0.623	0.602	1	0.0386
	0.02	14	0.553	0.237	34	0.674	0.529	0.989	0.0218
	0.03	14	0.486	0.195	52	0.692	0.476	0.953	0.0131
	0.04	14	0.439	0.168	76	0.702	0.438	0.896	0.0086
	0.05	14	0.405	0.149	102	0.706	0.408	0.823	0.0063
	0.06	14	0.377	0.134	134	0.711	0.383	0.740	0.0048
	0.07	14	0.353	0.121	172	0.713	0.361	0.646	0.0037
	0.08	14	0.331	0.110	218	0.716	0.340	0.540	0.0030
(0.1,0.7) or (0.3,0.9)	0.001	20	0.814	0.616	20	0.616	0.590	1	0
	0.01	20	0.670	0.364	30	0.636	0.518	0.999	0.0272
	0.02	20	0.578	0.295	48	0.677	0.463	0.979	0.0136
	0.03	20	0.523	0.260	72	0.697	0.426	0.931	0.0084
	0.04	20	0.488	0.238	98	0.705	0.401	0.866	0.0060
	0.05	20	0.462	0.223	126	0.708	0.382	0.792	0.0046
	0.06	20	0.442	0.211	154	0.712	0.367	0.712	0.0037
	0.07	20	0.424	0.200	186	0.714	0.353	0.627	0.0031
	0.08	20	0.407	0.189	222	0.716	0.338	0.534	0.0026
(0.2,0.8)	0.001	20	0.800	0.537	22	0.644	0.593	1	0.0537
	0.01	20	0.676	0.372	30	0.643	0.530	0.999	0.0271
	0.02	20	0.588	0.308	48	0.680	0.474	0.982	0.0133
	0.03	20	0.532	0.272	70	0.695	0.435	0.936	0.0085
	0.04	20	0.495	0.247	94	0.703	0.408	0.872	0.0062
	0.05	20	0.467	0.229	122	0.709	0.387	0.798	0.0047
	0.06	20	0.445	0.215	152	0.711	0.370	0.717	0.0038
	0.07	20	0.425	0.202	184	0.714	0.354	0.630	0.0031
	0.08	20	0.407	0.189	222	0.716	0.339	0.534	0.0026
(0.1,0.6) or (0.4,0.9)	0.001	28	0.804	0.572	28	0.572	0.491	1	0
	0.01	28	0.655	0.368	48	0.660	0.430	0.996	0.0146
	0.02	28	0.578	0.319	76	0.689	0.393	0.958	0.0077
	0.03	28	0.540	0.299	106	0.701	0.374	0.895	0.0052
	0.04	28	0.518	0.290	132	0.706	0.362	0.824	0.0040
	0.05	28	0.504	0.285	158	0.710	0.355	0.752	0.0033
	0.06	28	0.494	0.281	180	0.713	0.349	0.680	0.0028
	0.07	28	0.486	0.278	202	0.714	0.343	0.606	0.0025
	0.08	28	0.476	0.272	226	0.715	0.336	0.528	0.0022
(0.2,0.7) or (0.3,0.8)	0.001	30	0.803	0.558	30	0.558	0.494	1	0
	0.01	30	0.682	0.412	46	0.653	0.446	0.998	0.0151
	0.02	30	0.609	0.365	72	0.686	0.410	0.966	0.0076
	0.03	30	0.570	0.342	100	0.700	0.388	0.907	0.0051
	0.04	30	0.546	0.328	126	0.706	0.374	0.837	0.0039
	0.05	30	0.529	0.317	150	0.710	0.363	0.763	0.0033
	0.06	30	0.515	0.309	174	0.713	0.354	0.688	0.0028
	0.07	30	0.503	0.300	198	0.714	0.346	0.611	0.0025
	0.08	30	0.491	0.291	226	0.716	0.337	0.529	0.0022
(0.1,0.5) or (0.5,0.9)	0.001	40	0.781	0.488	42	0.566	0.392	1	0.0393
	0.01	40	0.627	0.358	80	0.675	0.343	0.985	0.0079
	0.02	40	0.573	0.335	124	0.697	0.326	0.918	0.0043
	0.03	40	0.553	0.334	160	0.706	0.322	0.841	0.0031
	0.04	40	0.546	0.339	182	0.709	0.324	0.769	0.0026
	0.05	40	0.545	0.346	200	0.712	0.327	0.703	0.0023
	0.06	40	0.545	0.353	210	0.713	0.331	0.642	0.0021
	0.07	40	0.546	0.359	220	0.715	0.334	0.584	0.0020
	0.08	40	0.545	0.362	230	0.716	0.334	0.522	0.0019
(0.2,0.6) or (0.4,0.8)	0.001	46	0.791	0.518	48	0.587	0.395	1	0.0345
	0.01	46	0.668	0.422	80	0.668	0.360	0.991	0.0072
	0.02	46	0.617	0.401	118	0.694	0.343	0.933	0.0041
	0.03	46	0.597	0.397	150	0.704	0.339	0.860	0.0030
	0.04	46	0.588	0.397	172	0.709	0.338	0.787	0.0025
	0.05	46	0.584	0.399	190	0.712	0.338	0.719	0.0022
	0.06	46	0.580	0.400	202	0.713	0.337	0.654	0.0020
	0.07	46	0.577	0.400	216	0.714	0.337	0.589	0.0018
	0.08	46	0.572	0.398	230	0.715	0.335	0.523	0.0017
(0.3,0.7)	0.001	48	0.793	0.524	50	0.558	0.396	1	0.0167
	0.01	48	0.678	0.438	80	0.665	0.365	0.992	0.0071
	0.02	48	0.629	0.419	118	0.695	0.349	0.938	0.0039
	0.03	48	0.610	0.415	146	0.704	0.344	0.866	0.0029
	0.04	48	0.601	0.415	168	0.709	0.342	0.794	0.0024
	0.05	48	0.595	0.415	186	0.711	0.341	0.724	0.0021
	0.06	48	0.591	0.415	200	0.714	0.340	0.658	0.0020
	0.07	48	0.586	0.413	214	0.715	0.338	0.591	0.0018
	0.08	48	0.580	0.409	230	0.715	0.335	0.523	0.0017
(0.1,0.4) or (0.6,0.9)	0.001	64	0.763	0.461	72	0.588	0.292	1	0.0159
	0.01	64	0.612	0.377	156	0.689	0.262	0.951	0.0034
	0.02	64	0.588	0.381	216	0.705	0.265	0.850	0.0021
	0.03	64	0.588	0.397	242	0.709	0.276	0.768	0.0018
	0.04	64	0.594	0.414	250	0.712	0.289	0.703	0.0016
	0.05	64	0.603	0.432	250	0.714	0.302	0.649	0.0015
	0.06	64	0.613	0.449	246	0.715	0.314	0.603	0.0015
	0.07	64	0.622	0.464	238	0.715	0.325	0.560	0.0014
	0.08	64	0.628	0.474	234	0.715	0.332	0.516	0.0014
(0.2,0.5) or (0.5,0.8)	0.001	78	0.776	0.491	84	0.584	0.296	1	0.0154
	0.01	78	0.654	0.441	158	0.686	0.277	0.964	0.0031
	0.02	78	0.635	0.450	210	0.703	0.282	0.872	0.0019
	0.03	78	0.635	0.465	232	0.709	0.292	0.791	0.0016
	0.04	78	0.640	0.480	238	0.711	0.302	0.724	0.0014
	0.05	78	0.647	0.493	240	0.713	0.312	0.666	0.0014
	0.06	78	0.653	0.504	238	0.714	0.321	0.615	0.0013
	0.07	78	0.658	0.514	234	0.715	0.328	0.566	0.0013
	0.08	78	0.661	0.520	234	0.716	0.332	0.517	0.0013
(0.3,0.6) or (0.4,0.7)	0.001	84	0.775	0.483	92	0.584	0.297	1	0.0126
	0.01	84	0.666	0.457	162	0.683	0.283	0.968	0.0029
	0.02	84	0.649	0.471	210	0.702	0.289	0.881	0.0018
	0.03	84	0.652	0.489	228	0.708	0.300	0.802	0.0015
	0.04	84	0.658	0.504	234	0.712	0.309	0.735	0.0014
	0.05	84	0.663	0.516	234	0.713	0.318	0.676	0.0013
	0.06	84	0.668	0.525	232	0.714	0.324	0.621	0.0013
	0.07	84	0.671	0.532	232	0.715	0.329	0.569	0.0012
	0.08	84	0.672	0.536	234	0.716	0.333	0.517	0.0012
(0.1,0.3) or (0.7,0.9)	0.001	124	0.736	0.453	152	0.614	0.194	1	0.0057
	0.01	124	0.625	0.434	346	0.703	0.193	0.866	0.0012
	0.02	124	0.636	0.470	374	0.710	0.216	0.750	0.0010
	0.03	124	0.654	0.501	358	0.712	0.239	0.680	0.0009
	0.04	124	0.671	0.528	334	0.714	0.261	0.631	0.0009
	0.05	124	0.687	0.553	306	0.714	0.281	0.594	0.0009
	0.06	124	0.701	0.575	280	0.715	0.300	0.565	0.0009
	0.07	124	0.714	0.594	258	0.715	0.317	0.538	0.0009
	0.08	124	0.725	0.610	240	0.716	0.330	0.510	0.0009
(0.2,0.4) or (0.6,0.8)	0.001	164	0.754	0.487	190	0.605	0.197	1	0.0045
	0.01	164	0.667	0.496	372	0.702	0.205	0.887	0.0010
	0.02	164	0.683	0.537	380	0.709	0.229	0.774	0.0008
	0.03	164	0.701	0.568	356	0.712	0.251	0.702	0.0007
	0.04	164	0.716	0.593	326	0.714	0.271	0.650	0.0007
	0.05	164	0.730	0.614	300	0.714	0.289	0.609	0.0007
	0.06	164	0.742	0.632	276	0.715	0.305	0.574	0.0007
	0.07	164	0.752	0.647	254	0.715	0.319	0.543	0.0008
	0.08	164	0.760	0.659	240	0.716	0.330	0.511	0.0008
(0.3,0.5) or (0.5,0.7)	0.001	186	0.755	0.487	214	0.605	0.198	1	0.0042
	0.01	186	0.679	0.513	394	0.700	0.210	0.896	0.0009
	0.02	186	0.699	0.560	390	0.709	0.236	0.787	0.0007
	0.03	186	0.719	0.594	356	0.712	0.259	0.715	0.0007
	0.04	186	0.735	0.620	324	0.713	0.278	0.662	0.0007
	0.05	186	0.748	0.640	294	0.714	0.294	0.619	0.0007
	0.06	186	0.759	0.656	272	0.715	0.308	0.581	0.0007
	0.07	186	0.768	0.669	252	0.715	0.321	0.546	0.0007
	0.08	186	0.774	0.679	238	0.716	0.331	0.511	0.0007
(0.4,0.6)	0.001	194	0.757	0.491	222	0.603	0.198	1	0.0040
	0.01	194	0.683	0.518	402	0.700	0.211	0.898	0.0009
	0.02	194	0.704	0.567	396	0.709	0.238	0.791	0.0007
	0.03	194	0.724	0.602	358	0.712	0.261	0.720	0.0007
	0.04	194	0.741	0.628	322	0.713	0.280	0.666	0.0007
	0.05	194	0.754	0.648	294	0.714	0.296	0.622	0.0007
	0.06	194	0.765	0.664	270	0.715	0.310	0.583	0.0007
	0.07	194	0.773	0.677	252	0.715	0.321	0.547	0.0007
	0.08	194	0.779	0.686	238	0.716	0.331	0.511	0.0007
(0.1,0.2) or (0.8,0.9)	0.001	398	0.683	0.470	676	0.675	0.098	0.981	0.0007
	0.01	398	0.712	0.584	794	0.712	0.141	0.708	0.0003
	0.02	398	0.750	0.642	618	0.714	0.179	0.626	0.0003
	0.03	398	0.775	0.679	504	0.715	0.211	0.586	0.0003
	0.04	398	0.794	0.706	424	0.715	0.239	0.562	0.0003
	0.05	398	0.809	0.728	364	0.715	0.265	0.544	0.0004
	0.06	398	0.822	0.747	316	0.716	0.288	0.530	0.0004
	0.07	398	0.833	0.763	276	0.716	0.310	0.518	0.0004
	0.08	398	0.843	0.777	244	0.716	0.328	0.505	0.0004
(0.2,0.3) or (0.7,0.8)	0.001	588	0.701	0.495	924	0.672	0.100	0.985	0.0005
	0.01	588	0.746	0.633	922	0.711	0.149	0.727	0.0002
	0.02	588	0.787	0.695	666	0.714	0.188	0.644	0.0002
	0.03	588	0.812	0.731	524	0.715	0.218	0.601	0.0003
	0.04	588	0.829	0.756	432	0.715	0.245	0.573	0.0003
	0.05	588	0.842	0.775	366	0.716	0.269	0.552	0.0003
	0.06	588	0.853	0.791	316	0.716	0.291	0.535	0.0003
	0.07	588	0.863	0.805	276	0.716	0.311	0.520	0.0003
	0.08	588	0.871	0.816	244	0.716	0.328	0.506	0.0003
(0.3,0.4) or (0.6,0.7)	0.001	712	0.704	0.500	1100	0.671	0.101	0.986	0.0004
	0.01	712	0.756	0.647	1034	0.711	0.153	0.736	0.0002
	0.02	712	0.800	0.713	714	0.714	0.193	0.654	0.0005
	0.03	712	0.825	0.751	544	0.714	0.224	0.611	0.0002
	0.04	712	0.843	0.776	440	0.715	0.250	0.581	0.0002
	0.05	712	0.856	0.795	368	0.715	0.273	0.559	0.0002
	0.06	712	0.867	0.810	314	0.716	0.294	0.540	0.0002
	0.07	712	0.875	0.823	274	0.716	0.312	0.523	0.0002
	0.08	712	0.882	0.833	244	0.716	0.329	0.506	0.0003
(0.4,0.5) or (0.5,0.6)	0.001	776	0.706	0.502	1190	0.670	0.101	0.986	0.0004
	0.01	776	0.760	0.652	1096	0.711	0.154	0.740	0.0002
	0.02	776	0.804	0.720	744	0.713	0.195	0.659	0.0002
	0.03	776	0.831	0.758	558	0.714	0.226	0.615	0.0002
	0.04	776	0.849	0.784	446	0.715	0.253	0.586	0.0002
	0.05	776	0.862	0.803	368	0.715	0.275	0.562	0.0002
	0.06	776	0.872	0.818	314	0.716	0.295	0.542	0.0002
	0.07	776	0.881	0.830	274	0.716	0.313	0.524	0.0002
	0.08	776	0.887	0.840	244	0.716	0.329	0.506	0.0002

**Table 5:** Sample scenarios assuming beta priors *p*(*π*_1_) and *p*(*π*_2_) where $\tau _{1}^{2}=0.001$. Hypothesized values of *π*_1_ and *π*_2_ set equal to *m*_1_ and *m*_2_, respectively, under the traditional design. Two-sided *α*=0.05 and 1−*β*=0.80 assumed

	($\tau _{1}^{2}=0.001$)	Traditional design			CEP design				
(*m*_1_,*m*_2_)	$\tau _{2}^{2}$	$\hat {N}$	Performance	CEP	*N* ^∗^	Performance	*E*(*π*_2_−*π*_1_|*π*_2_>*π*_1_)	*P*(*π*_2_>*π*_1_)	Marginal benefit
(0.1,0.9)	0.01	10	0.344	0.706	14	0.640	0.728	1	0.0740
	0.02	10	0.280	0.639	16	0.615	0.682	1	0.0559
	0.03	10	0.239	0.586	20	0.655	0.640	0.999	0.0416
	0.04	10	0.208	0.539	24	0.659	0.601	0.997	0.0322
	0.05	10	0.184	0.497	30	0.671	0.564	0.991	0.0244
	0.06	10	0.163	0.459	38	0.687	0.528	0.979	0.0187
	0.07	10	0.144	0.424	50	0.693	0.494	0.955	0.0137
	0.08	10	0.127	0.389	70	0.703	0.459	0.913	0.0096
(0.1,0.8)	0.01	14	0.439	0.748	16	0.586	0.656	1	0.0735
	0.02	14	0.389	0.697	20	0.623	0.624	1	0.0390
	0.03	14	0.353	0.652	24	0.651	0.592	0.999	0.0298
	0.04	14	0.324	0.611	28	0.667	0.561	0.997	0.0245
	0.05	14	0.299	0.574	34	0.674	0.533	0.990	0.0187
	0.06	14	0.278	0.540	44	0.687	0.505	0.975	0.0136
	0.07	14	0.259	0.509	56	0.695	0.480	0.950	0.0104
	0.08	14	0.241	0.480	72	0.703	0.455	0.910	0.0080
(0.1,0.7)	0.01	20	0.507	0.773	22	0.603	0.572	1	0.0481
	0.02	20	0.466	0.732	26	0.623	0.552	1	0.0262
	0.03	20	0.438	0.695	30	0.643	0.532	0.999	0.0205
	0.04	20	0.416	0.662	36	0.667	0.513	0.995	0.0157
	0.05	20	0.397	0.633	42	0.680	0.494	0.986	0.0129
	0.06	20	0.382	0.607	52	0.691	0.478	0.969	0.0097
	0.07	20	0.369	0.585	62	0.696	0.464	0.943	0.0078
	0.08	20	0.358	0.566	76	0.705	0.450	0.906	0.0062
(0.2,0.8)	0.01	20	0.430	0.734	24	0.601	0.561	1	0.0428
	0.02	20	0.382	0.676	30	0.641	0.528	0.999	0.0259
	0.03	20	0.349	0.628	38	0.666	0.499	0.995	0.0176
	0.04	20	0.324	0.589	48	0.681	0.473	0.982	0.0127
	0.05	20	0.305	0.557	62	0.693	0.452	0.960	0.0092
	0.06	20	0.289	0.530	76	0.699	0.434	0.927	0.0073
	0.07	20	0.276	0.508	96	0.706	0.418	0.881	0.0057
	0.08	20	0.264	0.488	118	0.710	0.403	0.821	0.0046
(0.1,0.6)	0.01	28	0.511	0.770	32	0.604	0.483	1	0.0231
	0.02	28	0.490	0.737	36	0.631	0.474	1	0.0175
	0.03	28	0.477	0.708	40	0.651	0.465	0.998	0.0145
	0.04	28	0.467	0.685	46	0.667	0.457	0.991	0.0111
	0.05	28	0.461	0.667	54	0.685	0.452	0.978	0.0086
	0.06	28	0.458	0.653	62	0.691	0.448	0.959	0.0069
	0.07	28	0.457	0.643	70	0.698	0.446	0.934	0.0057
	0.08	28	0.458	0.636	78	0.704	0.446	0.903	0.0049
(0.2,0.7)	0.01	30	0.479	0.750	36	0.608	0.477	1	0.0214
	0.02	30	0.447	0.702	44	0.649	0.458	0.999	0.0144
	0.03	30	0.426	0.664	54	0.671	0.441	0.991	0.0102
	0.04	30	0.411	0.636	66	0.686	0.427	0.974	0.0076
	0.05	30	0.401	0.615	78	0.695	0.417	0.947	0.0061
	0.06	30	0.395	0.599	92	0.702	0.410	0.912	0.0050
	0.07	30	0.391	0.587	106	0.706	0.404	0.868	0.0042
	0.08	30	0.388	0.578	122	0.711	0.400	0.816	0.0035
(0.1,0.5)	0.01	40	0.490	0.751	48	0.614	0.392	1	0.0155
	0.02	40	0.493	0.725	54	0.643	0.392	0.999	0.0107
	0.03	40	0.498	0.708	60	0.666	0.395	0.993	0.0084
	0.04	40	0.504	0.697	66	0.678	0.400	0.981	0.0067
	0.05	40	0.514	0.692	72	0.689	0.407	0.965	0.0055
	0.06	40	0.525	0.692	76	0.695	0.417	0.945	0.0047
	0.07	40	0.538	0.694	80	0.701	0.429	0.923	0.0041
	0.08	40	0.552	0.699	80	0.705	0.442	0.899	0.0038
(0.2,0.6)	0.01	46	0.487	0.742	56	0.624	0.388	1	0.0137
	0.02	46	0.475	0.704	68	0.659	0.380	0.996	0.0083
	0.03	46	0.471	0.679	82	0.678	0.376	0.981	0.0057
	0.04	46	0.472	0.665	94	0.691	0.376	0.956	0.0046
	0.05	46	0.477	0.658	106	0.698	0.379	0.925	0.0037
	0.06	46	0.484	0.655	114	0.704	0.384	0.890	0.0032
	0.07	46	0.493	0.655	120	0.707	0.390	0.851	0.0029
	0.08	46	0.502	0.658	126	0.711	0.396	0.810	0.0026
(0.3,0.7)	0.01	48	0.465	0.724	62	0.630	0.379	1	0.0118
	0.02	48	0.440	0.673	82	0.670	0.363	0.990	0.0068
	0.03	48	0.429	0.643	104	0.689	0.354	0.963	0.0046
	0.04	48	0.426	0.626	124	0.698	0.349	0.924	0.0036
	0.05	48	0.425	0.616	144	0.705	0.347	0.879	0.0029
	0.06	48	0.427	0.610	158	0.708	0.347	0.829	0.0026
	0.07	48	0.431	0.607	172	0.711	0.348	0.776	0.0023
	0.08	48	0.435	0.605	182	0.713	0.349	0.718	0.0021
(0.1,0.4)	0.01	64	0.497	0.735	82	0.635	0.300	0.999	0.0077
	0.02	64	0.519	0.720	92	0.667	0.312	0.992	0.0053
	0.03	64	0.540	0.717	98	0.680	0.328	0.977	0.0041
	0.04	64	0.563	0.722	102	0.691	0.346	0.960	0.0034
	0.05	64	0.585	0.730	100	0.696	0.366	0.942	0.0031
	0.06	64	0.608	0.741	96	0.700	0.388	0.925	0.0029
	0.07	64	0.632	0.754	90	0.703	0.412	0.909	0.0027
	0.08	64	0.656	0.768	84	0.706	0.437	0.895	0.0025
(0.2,0.5)	0.01	78	0.496	0.726	104	0.644	0.297	0.998	0.0057
	0.02	78	0.505	0.701	126	0.677	0.302	0.982	0.0036
	0.03	78	0.521	0.695	140	0.691	0.313	0.954	0.0027
	0.04	78	0.540	0.698	148	0.699	0.327	0.922	0.0023
	0.05	78	0.559	0.705	148	0.704	0.342	0.890	0.0021
	0.06	78	0.579	0.715	144	0.707	0.358	0.860	0.0019
	0.07	78	0.599	0.726	138	0.709	0.375	0.831	0.0018
	0.08	78	0.620	0.738	130	0.711	0.393	0.804	0.0018
(0.3,0.6)	0.01	84	0.474	0.708	120	0.652	0.291	0.997	0.0049
	0.02	84	0.477	0.677	156	0.684	0.290	0.970	0.0029
	0.03	84	0.489	0.668	180	0.697	0.297	0.928	0.0022
	0.04	84	0.504	0.669	194	0.704	0.305	0.882	0.0018
	0.05	84	0.520	0.674	198	0.707	0.315	0.838	0.0016
	0.06	84	0.536	0.681	198	0.710	0.325	0.795	0.0015
	0.07	84	0.551	0.689	194	0.712	0.336	0.753	0.0015
	0.08	84	0.566	0.698	188	0.713	0.346	0.712	0.0014
(0.1,0.3)	0.01	124	0.525	0.718	182	0.671	0.214	0.987	0.0025
	0.02	124	0.574	0.730	186	0.688	0.242	0.960	0.0018
	0.03	124	0.614	0.749	174	0.695	0.271	0.937	0.0016
	0.04	124	0.650	0.768	156	0.699	0.301	0.920	0.0016
	0.05	124	0.681	0.787	138	0.702	0.332	0.908	0.0015
	0.06	124	0.710	0.805	120	0.704	0.363	0.899	0.0015
	0.07	124	0.738	0.822	102	0.704	0.397	0.894	0.0015
	0.08	124	0.765	0.840	86	0.705	0.433	0.891	0.0016
(0.2,0.4)	0.01	164	0.524	0.711	254	0.678	0.210	0.979	0.0017
	0.02	164	0.565	0.718	272	0.695	0.233	0.934	0.0012
	0.03	164	0.602	0.735	260	0.702	0.258	0.895	0.0010
	0.04	164	0.635	0.753	236	0.706	0.283	0.864	0.0010
	0.05	164	0.665	0.771	208	0.708	0.309	0.841	0.0010
	0.06	164	0.692	0.789	182	0.710	0.335	0.822	0.0010
	0.07	164	0.718	0.805	156	0.710	0.362	0.809	0.0010
	0.08	164	0.742	0.822	134	0.711	0.389	0.798	0.0010
(0.3,0.5)	0.01	186	0.509	0.697	314	0.683	0.205	0.971	0.0014
	0.02	186	0.545	0.701	348	0.699	0.224	0.909	0.0010
	0.03	186	0.580	0.717	336	0.705	0.244	0.857	0.0008
	0.04	186	0.610	0.734	310	0.708	0.265	0.815	0.0008
	0.05	186	0.638	0.751	280	0.711	0.285	0.780	0.0008
	0.06	186	0.663	0.767	250	0.711	0.304	0.751	0.0008
	0.07	186	0.686	0.782	222	0.713	0.324	0.726	0.0008
	0.08	186	0.707	0.796	194	0.714	0.343	0.704	0.0008
(0.4,0.6)	0.01	194	0.495	0.684	354	0.686	0.200	0.962	0.0012
	0.02	194	0.526	0.685	406	0.702	0.214	0.885	0.0008
	0.03	194	0.556	0.698	402	0.707	0.229	0.820	0.0007
	0.04	194	0.582	0.712	382	0.710	0.244	0.767	0.0007
	0.05	194	0.605	0.726	354	0.712	0.258	0.722	0.0007
	0.06	194	0.626	0.739	326	0.713	0.272	0.682	0.0007
	0.07	194	0.645	0.751	300	0.714	0.284	0.646	0.0007
	0.08	194	0.662	0.763	274	0.715	0.296	0.610	0.0007
(0.1,0.2)	0.01	398	0.626	0.752	562	0.701	0.145	0.897	0.0005
	0.02	398	0.703	0.799	402	0.705	0.190	0.870	0.0005
	0.03	398	0.751	0.830	302	0.706	0.230	0.861	0.0005
	0.04	398	0.786	0.853	234	0.706	0.268	0.861	0.0005
	0.05	398	0.814	0.872	184	0.707	0.305	0.864	0.0005
	0.06	398	0.837	0.888	146	0.707	0.343	0.870	0.0005
	0.07	398	0.859	0.903	116	0.707	0.384	0.878	0.0005
	0.08	398	0.879	0.917	88	0.706	0.429	0.887	0.0006
(0.2,0.3)	0.01	588	0.623	0.747	874	0.704	0.140	0.864	0.0003
	0.02	588	0.697	0.792	630	0.708	0.180	0.813	0.0003
	0.03	588	0.743	0.823	470	0.710	0.216	0.791	0.0003
	0.04	588	0.778	0.846	362	0.711	0.250	0.782	0.0003
	0.05	588	0.806	0.865	284	0.711	0.283	0.779	0.0003
	0.06	588	0.829	0.881	224	0.711	0.316	0.780	0.0003
	0.07	588	0.849	0.895	176	0.711	0.350	0.785	0.0003
	0.08	588	0.868	0.909	138	0.711	0.386	0.793	0.0004
(0.3,0.4)	0.01	712	0.614	0.739	1126	0.706	0.136	0.841	0.0002
	0.02	712	0.684	0.783	830	0.710	0.171	0.774	0.0002
	0.03	712	0.730	0.813	628	0.711	0.202	0.740	0.0002
	0.04	712	0.764	0.836	488	0.712	0.231	0.720	0.0002
	0.05	712	0.791	0.854	388	0.713	0.259	0.708	0.0002
	0.06	712	0.814	0.870	310	0.713	0.286	0.701	0.0003
	0.07	712	0.834	0.884	250	0.713	0.313	0.697	0.0003
	0.08	712	0.852	0.896	200	0.714	0.341	0.697	0.0003
(0.4,0.5)	0.01	776	0.605	0.732	1298	0.707	0.132	0.823	0.0002
	0.02	776	0.672	0.773	984	0.711	0.163	0.742	0.0002
	0.03	776	0.715	0.802	762	0.712	0.189	0.698	0.0002
	0.04	776	0.747	0.824	608	0.713	0.213	0.668	0.0002
	0.05	776	0.773	0.841	494	0.714	0.234	0.646	0.0002
	0.06	776	0.794	0.856	406	0.714	0.255	0.629	0.0002
	0.07	776	0.813	0.869	338	0.715	0.275	0.614	0.0002
	0.08	776	0.829	0.880	282	0.715	0.294	0.602	0.0002

**Table 6:** Sample scenarios assuming beta priors *p*(*π*_1_) and *p*(*π*_2_) where $\tau _{1}^{2}=0.08$. Hypothesized values of *π*_1_ and *π*_2_ set equal to *m*_1_ and *m*_2_, respectively, under the traditional design. Two-sided *α*=0.05 and 1−*β*=0.80 assumed

	($\tau _{1}^{2}=0.08$)	Traditional design			CEP design				
(*m*_1_,*m*_2_)	$\tau _{2}^{2}$	$\hat {N}$	Performance	CEP	*N* ^∗^	Performance	*E*(*π*_2_−*π*_1_|*π*_2_>*π*_1_)	*P*(*π*_2_>*π*_1_)	Marginal benefit
(0.1,0.9)	0.001	10	0.127	0.389	70	0.703	0.459	0.913	0.0096
	0.01	10	0.097	0.356	88	0.707	0.435	0.861	0.0078
	0.02	10	0.083	0.335	102	0.709	0.417	0.815	0.0068
	0.03	10	0.075	0.319	118	0.711	0.402	0.774	0.0059
	0.04	10	0.069	0.306	132	0.712	0.389	0.734	0.0053
	0.05	10	0.064	0.294	148	0.713	0.378	0.693	0.0047
	0.06	10	0.060	0.283	166	0.713	0.366	0.650	0.0042
	0.07	10	0.056	0.273	186	0.714	0.355	0.602	0.0037
(0.1,0.8)	0.001	14	0.147	0.399	114	0.710	0.407	0.825	0.0056
	0.01	14	0.134	0.387	124	0.710	0.396	0.794	0.0052
	0.02	14	0.128	0.377	134	0.711	0.386	0.760	0.0049
	0.03	14	0.124	0.368	146	0.712	0.378	0.728	0.0045
	0.04	14	0.121	0.360	158	0.713	0.370	0.695	0.0041
	0.05	14	0.118	0.353	170	0.713	0.362	0.661	0.0038
	0.06	14	0.116	0.346	184	0.714	0.355	0.625	0.0035
	0.07	14	0.113	0.339	198	0.715	0.348	0.586	0.0033
(0.1,0.7)	0.001	20	0.174	0.415	172	0.713	0.355	0.731	0.0035
	0.01	20	0.172	0.414	176	0.713	0.353	0.712	0.0035
	0.02	20	0.174	0.413	182	0.713	0.350	0.691	0.0033
	0.03	20	0.177	0.412	188	0.714	0.348	0.669	0.0032
	0.04	20	0.180	0.411	192	0.714	0.346	0.646	0.0031
	0.05	20	0.183	0.410	200	0.715	0.344	0.622	0.0030
	0.06	20	0.185	0.409	206	0.714	0.342	0.596	0.0028
	0.07	20	0.187	0.408	214	0.715	0.340	0.567	0.0027
(0.2,0.8)	0.001	20	0.264	0.488	118	0.710	0.403	0.821	0.0046
	0.01	20	0.247	0.475	128	0.711	0.393	0.789	0.0043
	0.02	20	0.233	0.462	138	0.712	0.384	0.755	0.0041
	0.03	20	0.223	0.451	150	0.712	0.375	0.723	0.0038
	0.04	20	0.215	0.442	162	0.713	0.367	0.690	0.0035
	0.05	20	0.208	0.433	174	0.713	0.360	0.656	0.0033
	0.06	20	0.201	0.424	188	0.714	0.353	0.620	0.0031
	0.07	20	0.195	0.416	202	0.715	0.346	0.580	0.0029
(0.1,0.6)	0.001	28	0.193	0.426	252	0.715	0.303	0.632	0.0023
	0.01	28	0.199	0.431	250	0.715	0.307	0.623	0.0023
	0.02	28	0.209	0.437	246	0.715	0.311	0.613	0.0023
	0.03	28	0.220	0.443	244	0.715	0.316	0.601	0.0023
	0.04	28	0.231	0.449	240	0.715	0.320	0.589	0.0023
	0.05	28	0.241	0.456	238	0.715	0.324	0.576	0.0023
	0.06	28	0.252	0.463	234	0.715	0.328	0.562	0.0022
	0.07	28	0.262	0.469	230	0.715	0.332	0.546	0.0022
(0.2,0.7)	0.001	30	0.302	0.510	178	0.713	0.352	0.725	0.0028
	0.01	30	0.298	0.507	182	0.713	0.350	0.706	0.0027
	0.02	30	0.295	0.504	186	0.714	0.348	0.685	0.0027
	0.03	30	0.293	0.502	192	0.714	0.346	0.663	0.0026
	0.04	30	0.292	0.499	198	0.714	0.344	0.640	0.0025
	0.05	30	0.291	0.497	204	0.714	0.342	0.616	0.0024
	0.06	30	0.291	0.495	210	0.715	0.340	0.590	0.0024
	0.07	30	0.291	0.493	218	0.715	0.338	0.561	0.0023
(0.1,0.5)	0.001	40	0.213	0.437	364	0.716	0.253	0.531	0.0016
	0.01	40	0.226	0.448	350	0.716	0.262	0.530	0.0016
	0.02	40	0.244	0.461	334	0.716	0.272	0.529	0.0016
	0.03	40	0.264	0.474	318	0.716	0.282	0.528	0.0016
	0.04	40	0.283	0.488	300	0.715	0.293	0.527	0.0017
	0.05	40	0.303	0.502	284	0.716	0.303	0.526	0.0017
	0.06	40	0.323	0.516	266	0.716	0.313	0.525	0.0017
	0.07	40	0.342	0.530	248	0.716	0.324	0.523	0.0018
(0.2,0.6)	0.001	46	0.343	0.536	258	0.715	0.301	0.625	0.0018
	0.01	46	0.347	0.539	256	0.715	0.305	0.617	0.0018
	0.02	46	0.352	0.543	252	0.715	0.309	0.606	0.0018
	0.03	46	0.359	0.547	250	0.715	0.314	0.595	0.0017
	0.04	46	0.366	0.552	246	0.715	0.318	0.583	0.0017
	0.05	46	0.373	0.556	242	0.715	0.322	0.570	0.0017
	0.06	46	0.382	0.562	238	0.715	0.326	0.556	0.0017
	0.07	46	0.390	0.567	234	0.716	0.330	0.540	0.0017
(0.3,0.7)	0.001	48	0.435	0.605	182	0.713	0.349	0.718	0.0021
	0.01	48	0.431	0.602	186	0.713	0.347	0.700	0.0020
	0.02	48	0.427	0.599	192	0.714	0.345	0.679	0.0020
	0.03	48	0.423	0.596	196	0.714	0.343	0.657	0.0020
	0.04	48	0.419	0.592	202	0.714	0.342	0.634	0.0019
	0.05	48	0.416	0.589	208	0.715	0.340	0.610	0.0019
	0.06	48	0.414	0.586	214	0.715	0.338	0.584	0.0018
	0.07	48	0.411	0.583	222	0.715	0.336	0.555	0.0017
(0.1,0.4)	0.001	64	0.258	0.468	532	0.716	0.202	0.428	0.0010
	0.01	64	0.281	0.486	494	0.716	0.217	0.435	0.0010
	0.02	64	0.308	0.506	452	0.716	0.234	0.444	0.0011
	0.03	64	0.336	0.526	412	0.716	0.250	0.454	0.0011
	0.04	64	0.363	0.546	374	0.716	0.267	0.464	0.0011
	0.05	64	0.391	0.566	338	0.716	0.283	0.475	0.0012
	0.06	64	0.418	0.586	302	0.716	0.299	0.487	0.0013
	0.07	64	0.446	0.607	268	0.716	0.316	0.501	0.0013
(0.2,0.5)	0.001	78	0.404	0.577	374	0.716	0.251	0.524	0.0011
	0.01	78	0.414	0.585	358	0.716	0.260	0.523	0.0011
	0.02	78	0.427	0.594	342	0.716	0.270	0.522	0.0011
	0.03	78	0.441	0.604	326	0.716	0.281	0.521	0.0011
	0.04	78	0.455	0.615	308	0.716	0.291	0.521	0.0011
	0.05	78	0.470	0.625	290	0.716	0.301	0.520	0.0012
	0.06	78	0.486	0.637	272	0.716	0.312	0.519	0.0012
	0.07	78	0.503	0.649	254	0.716	0.322	0.518	0.0012
(0.3,0.6)	0.001	84	0.497	0.647	266	0.715	0.299	0.618	0.0012
	0.01	84	0.501	0.649	264	0.715	0.303	0.609	0.0012
	0.02	84	0.505	0.652	260	0.715	0.307	0.599	0.0012
	0.03	84	0.509	0.655	256	0.715	0.311	0.588	0.0012
	0.04	84	0.514	0.658	252	0.715	0.316	0.576	0.0012
	0.05	84	0.519	0.661	248	0.715	0.320	0.564	0.0012
	0.06	84	0.524	0.665	244	0.715	0.324	0.550	0.0012
	0.07	84	0.530	0.668	240	0.716	0.328	0.534	0.0012
(0.1,0.3)	0.001	124	0.339	0.526	808	0.717	0.153	0.323	0.0006
	0.01	124	0.376	0.554	708	0.717	0.176	0.341	0.0006
	0.02	124	0.414	0.582	612	0.717	0.200	0.361	0.0006
	0.03	124	0.450	0.608	530	0.717	0.223	0.382	0.0007
	0.04	124	0.483	0.632	458	0.717	0.245	0.404	0.0007
	0.05	124	0.515	0.656	394	0.717	0.266	0.428	0.0007
	0.06	124	0.546	0.679	338	0.717	0.287	0.452	0.0008
	0.07	124	0.578	0.702	286	0.716	0.308	0.480	0.0009
(0.2,0.4)	0.001	164	0.502	0.647	546	0.717	0.201	0.420	0.0006
	0.01	164	0.519	0.659	506	0.716	0.216	0.428	0.0006
	0.02	164	0.537	0.672	464	0.717	0.233	0.437	0.0006
	0.03	164	0.557	0.686	422	0.717	0.249	0.447	0.0006
	0.04	164	0.576	0.700	382	0.716	0.265	0.457	0.0006
	0.05	164	0.596	0.715	344	0.716	0.282	0.469	0.0007
	0.06	164	0.617	0.730	308	0.716	0.298	0.482	0.0007
	0.07	164	0.638	0.745	272	0.716	0.314	0.495	0.0007
(0.3,0.5)	0.001	186	0.597	0.716	384	0.716	0.249	0.516	0.0006
	0.01	186	0.605	0.721	368	0.716	0.258	0.515	0.0006
	0.02	186	0.614	0.728	352	0.716	0.269	0.515	0.0006
	0.03	186	0.623	0.735	334	0.716	0.279	0.514	0.0006
	0.04	186	0.633	0.742	314	0.716	0.289	0.514	0.0006
	0.05	186	0.644	0.749	296	0.716	0.299	0.513	0.0007
	0.06	186	0.655	0.757	278	0.716	0.310	0.513	0.0007
	0.07	186	0.667	0.766	258	0.716	0.320	0.512	0.0007
(0.4,0.6)	0.001	194	0.662	0.763	274	0.715	0.296	0.610	0.0007
	0.01	194	0.664	0.764	270	0.715	0.301	0.602	0.0007
	0.02	194	0.667	0.766	266	0.715	0.305	0.592	0.0007
	0.03	194	0.669	0.768	262	0.715	0.309	0.581	0.0007
	0.04	194	0.672	0.770	258	0.715	0.314	0.569	0.0007
	0.05	194	0.675	0.772	254	0.715	0.318	0.557	0.0007
	0.06	194	0.679	0.774	250	0.716	0.322	0.543	0.0007
	0.07	194	0.682	0.777	244	0.716	0.326	0.528	0.0007
(0.1,0.2)	0.001	398	0.506	0.648	1346	0.717	0.104	0.218	0.0002
	0.01	398	0.563	0.691	978	0.714	0.139	0.247	0.0003
	0.02	398	0.606	0.720	814	0.717	0.172	0.286	0.0003
	0.03	398	0.642	0.746	660	0.717	0.201	0.319	0.0003
	0.04	398	0.673	0.768	542	0.717	0.227	0.352	0.0003
	0.05	398	0.700	0.788	450	0.717	0.253	0.386	0.0003
	0.06	398	0.726	0.807	372	0.717	0.277	0.422	0.0004
	0.07	398	0.751	0.825	304	0.716	0.302	0.460	0.0004
(0.2,0.3)	0.001	588	0.667	0.765	828	0.717	0.152	0.316	0.0002
	0.01	588	0.691	0.783	694	0.714	0.175	0.330	0.0002
	0.02	588	0.709	0.794	624	0.717	0.199	0.355	0.0002
	0.03	588	0.728	0.808	540	0.717	0.222	0.376	0.0002
	0.04	588	0.747	0.821	466	0.717	0.243	0.398	0.0002
	0.05	588	0.764	0.834	402	0.717	0.265	0.422	0.0003
	0.06	588	0.781	0.846	344	0.717	0.286	0.447	0.0003
	0.07	588	0.799	0.858	292	0.716	0.307	0.474	0.0003
(0.3,0.4)	0.001	712	0.748	0.822	560	0.717	0.200	0.413	0.0002
	0.01	712	0.761	0.832	502	0.715	0.215	0.416	0.0002
	0.02	712	0.767	0.836	474	0.717	0.231	0.430	0.0002
	0.03	712	0.778	0.843	432	0.716	0.248	0.440	0.0002
	0.04	712	0.788	0.851	390	0.716	0.264	0.451	0.0002
	0.05	712	0.799	0.859	352	0.716	0.280	0.462	0.0002
	0.06	712	0.810	0.866	314	0.716	0.296	0.475	0.0002
	0.07	712	0.821	0.874	278	0.716	0.312	0.490	0.0002
(0.4,0.5)	0.001	776	0.797	0.857	394	0.716	0.247	0.508	0.0002
	0.01	776	0.803	0.863	368	0.714	0.257	0.503	0.0002
	0.02	776	0.806	0.863	360	0.716	0.267	0.507	0.0002
	0.03	776	0.811	0.867	342	0.716	0.277	0.507	0.0002
	0.04	776	0.816	0.871	322	0.716	0.287	0.507	0.0002
	0.05	776	0.822	0.875	302	0.716	0.298	0.506	0.0002
	0.06	776	0.827	0.879	284	0.716	0.308	0.506	0.0002
	0.07	776	0.834	0.883	264	0.716	0.318	0.506	0.0002

**Table 7:** Sample scenarios assuming uniform priors *p*(*π*_1_) and *p*(*π*_2_) where $\tau _{1}^{2}=\tau _{2}^{2}$. Hypothesized values of *π*_1_ and *π*_2_ set equal to *μ*_1_ and *μ*_2_, respectively, under the traditional design. Two-sided *α*=0.05 and 1−*β*=0.80 assumed

		Traditional design			CEP design				
(*μ*_1_,*μ*_2_)	$\tau _{1}^{2}=\tau _{2}^{2}$	$\hat {N}$	Performance	CEP	*N* ^∗^	Performance	*E*(*π*_2_−*π*_1_|*π*_2_>*π*_1_)	*P*(*π*_2_>*π*_1_)	Marginal benefit
(0.1,0.9)	0.001	10	0.638	0.826	10	0.638	0.800	1	0
(0.1,0.8) or (0.2,0.9)	0.001	14	0.643	0.824	14	0.643	0.700	1	0
(0.1,0.7) or (0.3,0.9)	0.001	20	0.680	0.831	20	0.680	0.600	1	0
(0.2,0.8)	0.001	20	0.593	0.811	20	0.593	0.600	1	0
	0.01	20	0.556	0.775	22	0.615	0.600	1	0.030
(0.1,0.6)or (0.4,0.9)	0.001	28	0.633	0.822	28	0.633	0.500	1	0
(0.2,0.7)or (0.3,0.8)	0.001	30	0.599	0.812	30	0.599	0.500	1	0
	0.01	30	0.554	0.763	36	0.650	0.500	1	0.016
(0.1,0.5) or (0.5,0.9)	0.001	40	0.551	0.801	40	0.551	0.400	1	0
(0.2,0.6) or (0.4,0.8)	0.001	46	0.553	0.800	46	0.553	0.400	1	0
	0.01	46	0.535	0.735	62	0.663	0.400	1	0.008
(0.3,0.7)	0.001	48	0.555	0.800	48	0.555	0.400	1	0
	0.01	48	0.536	0.735	64	0.660	0.400	1	0.008
	0.02	48	0.549	0.711	80	0.694	0.407	0.983	0.005
	0.03	48	0.575	0.718	82	0.702	0.427	0.945	0.004
(0.1,0.4) or (0.6,0.9)	0.001	64	0.523	0.787	68	0.589	0.300	1	0.017
(0.2,0.5) or (0.5,0.8)	0.001	78	0.521	0.786	82	0.581	0.300	1	0.015
	0.01	78	0.526	0.704	130	0.689	0.303	0.991	0.003
(0.3,0.6) or (0.4,0.7)	0.001	84	0.506	0.781	90	0.591	0.300	1	0.014
	0.01	84	0.521	0.701	142	0.690	0.303	0.991	0.003
	0.02	84	0.566	0.712	150	0.704	0.330	0.925	0.002
	0.03	84	0.603	0.731	140	0.709	0.357	0.875	0.002
(0.1,0.3) or (0.7,0.9)	0.001	124	0.510	0.764	140	0.600	0.200	1	0.006
(0.2,0.4) or (0.6,0.8)	0.001	164	0.512	0.764	184	0.603	0.200	1	0.005
	0.01	164	0.566	0.713	292	0.703	0.224	0.911	0.001
(0.3,0.5) or (0.5,0.7)	0.001	186	0.504	0.761	210	0.603	0.200	1	0.004
	0.01	186	0.562	0.710	336	0.704	0.224	0.911	0.001
	0.02	186	0.626	0.745	284	0.709	0.263	0.825	0.001
	0.03	186	0.669	0.772	236	0.710	0.295	0.778	0.001
(0.4,0.6)	0.001	194	0.504	0.761	220	0.607	0.200	1	0.004
	0.01	194	0.562	0.709	350	0.704	0.224	0.911	0.001
	0.02	194	0.625	0.745	296	0.708	0.263	0.825	0.001
	0.03	194	0.668	0.771	248	0.711	0.295	0.778	0.001
	0.04	194	0.700	0.792	210	0.712	0.323	0.747	0.001
	0.05	194	0.725	0.809	194	0.725	0.349	0.725	0

## Abbreviations

CEP: Conditional expected power
EP: Expected power
